# The dependence on ammonia pretreatment of N−O activation by Co(II) sites in zeolites: a DFT and ab initio molecular dynamics study

**DOI:** 10.1007/s00894-017-3322-z

**Published:** 2017-04-13

**Authors:** E. Broclawik, K. Góra-Marek, M. Radoń, T. Bučko, A. Stępniewski

**Affiliations:** 1 Jerzy Haber Institute of Catalysis and Surface Chemistry Polish Academy of Sciences, Niezapominajek 8, 30-239 Krakow, Poland; 2 Faculty of Chemistry, Jagiellonian University, Ingardena 3, 30-060 Krakow, Poland; 3 Department of Physical and Theoretical Chemistry, Faculty of Natural Sciences, Comenius University, Mlynská Dolina SK-84215 Bratislava, Slovakia

**Keywords:** Co(II) zeolites, DFT cluster and periodic modeling, Donor ligands, NH_3_ co-adsorption, NO stretching frequencies, IR spectra

## Abstract

This work is focused on the donor properties of cobalt-exchanged cationic sites in zeolites. It is based on cluster and periodic density functional theory modeling for relevant {[Co(II)(NH_3_)_n_]–NO} adducts, where Co(II) means a cobalt cation embedded either in a periodic model of chabasite (CHA) zeolite or in model clusters. NO stretching frequencies were derived from MD trajectories and compared to harmonic values from cluster calculations. By relating calculated NO frequencies to experimental FTIR spectra, it was shown that the forms of {Co(II)-NO} adducts comprising three or four ammonia co-ligands dominate the spectrum taken in ammonia-saturation conditions while forms with two NH_3_ ligands prevail under intermediate ammonia saturation. Finally, this work confirms the critical dependence of Co(II) activation ability towards NO upon the center donor properties, reinforced by ligation of strong donor ammonia ligands. However, strongly bound ligands appear also to compete with interaction of the center with the electron-rich framework, and a balance must be observed to maintain optimal activation ability.

**Electronic supplementary material:**

The online version of this article (doi:10.1007/s00894-017-3322-z) contains supplementary material, which is available to authorized users.

**Graphical abstract:**
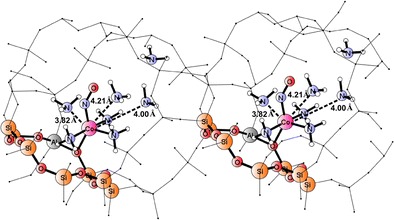
A snapshot from MD trajectory showing a fragment of periodic framework with twoCo(II)–NO centers, bound to one framework oxygen and strongly coordinating three ammonia ligands with four others forming the second coordination sphere

## Introduction

This paper is based on cluster and periodic density functional theory (DFT) modeling, focused on the donor properties of cobalt-exchanged cationic sites in zeolites. The direct goal of our calculations was to investigate the mechanism of enhancing the ability of Co(II) sites in zeolites to activate the NO bond by ammonia co-adsorption. In addition, we attempted to contribute to the fundamental problem of divalent cation siting in zeolites with a range of topologies and Si/Al ratios. In this respect, our study addresses the dependence of bonding of the Co^II^ cation with framework oxygens in the chabasite (CHA) zeolite, and its dependence upon the number of donor ammonia molecules, gradually saturating the first coordination sphere of the Co^II^ center containing bound NO ligand. Our former cluster DFT and correlated wavefunction results are supplemented by periodic DFT modeling and ab initio molecular dynamics (MD) simulations to introduce some preliminary insights into equilibrium of adsorption-desorption steps of NH_3_ molecules on Co(II) centers bound with NO, placed inside CHA channels under ambient conditions.

Nitric oxide ligand was selected because nitrogen–oxygen (N–O) stretching vibration modes frequently serve as key probes to study transition-metal active sites in molecular sieves by FTIR spectroscopy, with respect to their properties and catalytic activity. The second ligating agent, ammonia donor molecules are both a good source of electrons to increase donating ability of a cobalt site, and may serve themselves as a reducing agent in selective reduction processes (SCR) for NO abatement. The other active partner of the investigated system, the transition-metal active site in a molecular sieve, is itself of unremitting interest since metal-promoted, zeolite-based materials were found to be active for ammonia-selective catalytic reduction (NH_3_-SCR), a technology widely employed for NOx abatement [1, 2]. Here, Cu-exchanged SSZ-13 zeolites, based on CHA topology, have recently proven particularly appropriate for the NH_3_-SCR process, with exceptional activity and selectivity over a wide temperature window [3, 4]. Cobalt-exchanged zeolites were also found to be active in other important processes, especially in selective catalytic reduction with ammonia [5–7] or hydrocarbons (in particular SCR with methane) [8–12]. The distribution of Al T sites in a zeolite in the context of the exchange cation siting, and its impact on the SCR of NO by NH_3_ has already long been intensely studied experimentally by the group of Sobalik [13–15]. The topic has been indicated crucial to understanding the behavior of the active site in the reaction conditions, the role of substrates with respect to the site operando properties, and their impact on the activity of metallo-zeolites in redox N_2_O/NOx reactions The same subject has been addressed by Schneider et al. both experimentally and theoretically (see [16] and references therein); condition-dependent speciation and dynamics of exchanged Cu cations was shown to be extremely important for catalytic reactivity of Cu-SSZ-13 zeolites.

We have already long been involved in studies on donor properties of transition metal exchanged cations in zeolites, with a focus on their impact on the activation ability of ligands, especially towards NO [17–23]. In the last decade, our studies have centered on the proper quantum chemical description of electron density and its flow between the bound subsystems in the course of NO adsorption; special attention was paid recently to non-innocent systems, the Co(II) center bound with NO being a prominent case-study. It is worth noting that, to the best of our knowledge, there have been very limited theoretical studies on the last topic [24–26] because of the intricate nature of the electronic structure underlying the formation of the Co–NO bond, and the consequent high demands upon QM methodology. In our last two papers [22, 23] we partly resolved the issue of electron transfer channels between the cobalt center and the NO ligand (both odd-electron systems): we showed that, like other typical π-bound ligands conforming with the Chatt-Duncanson concept, NO may accept electrons from metal d orbitals to its empty π* orbital (activation) via a π-back-donation channel. However, since the NO ligand has one π* orbital partly occupied, it may either donate electrons to metal d_π_ orbitals via π-donation channels, or share the π* electron to form a covalent, partly polarized bond; the latter processes may contribute not only to NO bond deactivation but also to its activation. Thus, there are three major factors responsible for the donation-driven part of the NO activation mechanism, each of them strongly dependent on the electronic state of the adduct, its geometry, and the zeolite environment, which makes full analysis of this mechanism complicated, and predicting any final effect a hard task, in the case of Co(II) site.

The additional factor influencing the charge transfer processes under question comes from adsorption of other donor ligands to the metal center. In our recent papers we have suggested that, even though NH_3_ is a strong donor ligand, the bonding of consecutive ammonia molecules loosens the bonding of cobalt to electronegative framework oxygens in the zeolite fragment, being itself a strong electron donor. Therefore, the delicate balance between the number of NH_3_ molecules and the strength of bonding to framework oxygens must be obeyed to keep optimum electron transfer to NO and enforce its activation. It seems thus clear that no single descriptor can be defined to quantify the activity of the site towards weakening the NO bond. We have already proposed the relative contribution of the {Co^III^ – NO^−^} valence bond form to the total wave function of the complex (within multiconfiguration WF approach) as an effective theoretical measure of the electronic, donation-driven factor triggering NO activation. The experimentally oriented signature of activation is the calculated red-shift of NO stretching frequency, and our results confirmed that the two factors change in line for all studied complexes, and correlate well with IR experiments.

Small cluster models used in our former papers for modeling cobalt sites in an arbitrary zeolite also proved suitable for advanced correlated quantum chemical modeling, as they correctly predicted the two already introduced descriptors of Co(II) activity; they were also roughly validated by preliminary periodic calculations [23]. Unfortunately, they could neither discern zeolite frameworks with various topologies nor provide any information on the dynamic equilibria for {[Co(II)(NH_3_)_n_]–NO} systems in any actual zeolite. In this paper, we carefully arrange and append our former results with calculations for extended cluster models (inferred from periodic systems) in order to gain more information on the interplay of NH_3_ co-ligands with the cobalt center and of framework oxygens (representing the other, generalized, donor ligand), either competing or synergetically. In addition, we report preliminary ab initio molecular dynamics simulations for a periodic CHA framework containing Co^II^ compensating cations, with bound NO and a selected number of NH_3_ ligands. MD simulations were analyzed in terms of the dynamics of {[Co(II)(NH_3_)_n_]–NO} systems. The trajectories also serve to provide dynamics as average descriptors of the cobalt bonding to NH_3_ ligands and to framework oxygens. Anharmonic NO spectra were calculated from a Fourier transform of the auto-correlation function, filtered for NO vibrations; they not only validate cluster models but also allow deeper insight into the actual IR spectra.

Finally, we propose an enriched re-interpretation of in situ FTIR spectra reported for a selection of Co^II^-exchanged zeolites, taken under two distinct experimental conditions: zeolite pre-treated with doses of adsorbed ammonia corresponding either to an average of three NH_3_ molecules per Co(II) site (i), or to 4.5 ammonia ligands bound to cobalt (ii). The comparison of experimental NO stretching frequencies with those calculated for relevant {[Co(II)(NH_3_)_n_]–NO} adducts, both in a periodic model of CHA zeolite and in model clusters, reveal that NO stretching frequencies extracted from ab initio MD are in line with those obtained from Hessian diagonalization for cluster models. In general, anharmonic frequencies are closer to average experimental values registered for MOR, FER and BEA zeolites than their cluster counterparts; moreover, the proposal for their assignment was further enriched and modified with respect to former calculations. Careful analysis of trends in calculated and measured NO frequencies leads to the conclusion that the complexes accommodating two ammonia ligands (embedded either in periodic framework or in appropriate cluster models) mimic reasonably well the IR spectra taken below saturation limit with ammonia (i). On the contrary, our extended calculations indicate that, for fully ammonia-saturated zeolites (ii), the most abundant cobalt adducts (showing the highest red-shift of NO frequency) are found to bind three NH_3_ ligands to the Co(II)-NO core, being still linked to the framework. The inspection of MD trajectories (obtained under ambient simulation temperature and excess number of NH_3_) suggests that the formation of four-ammonia adducts requires breaking the bonding with framework oxygens and is registered less frequently while the binding of the fifth NH_3_ ligand in a realistic zeolite model appears much less probable.

## Modeling methodologies

### Static DFT and correlated wavefunction methods, cluster models

#### Cluster UDFT computations

**Fig. 1a,b:**
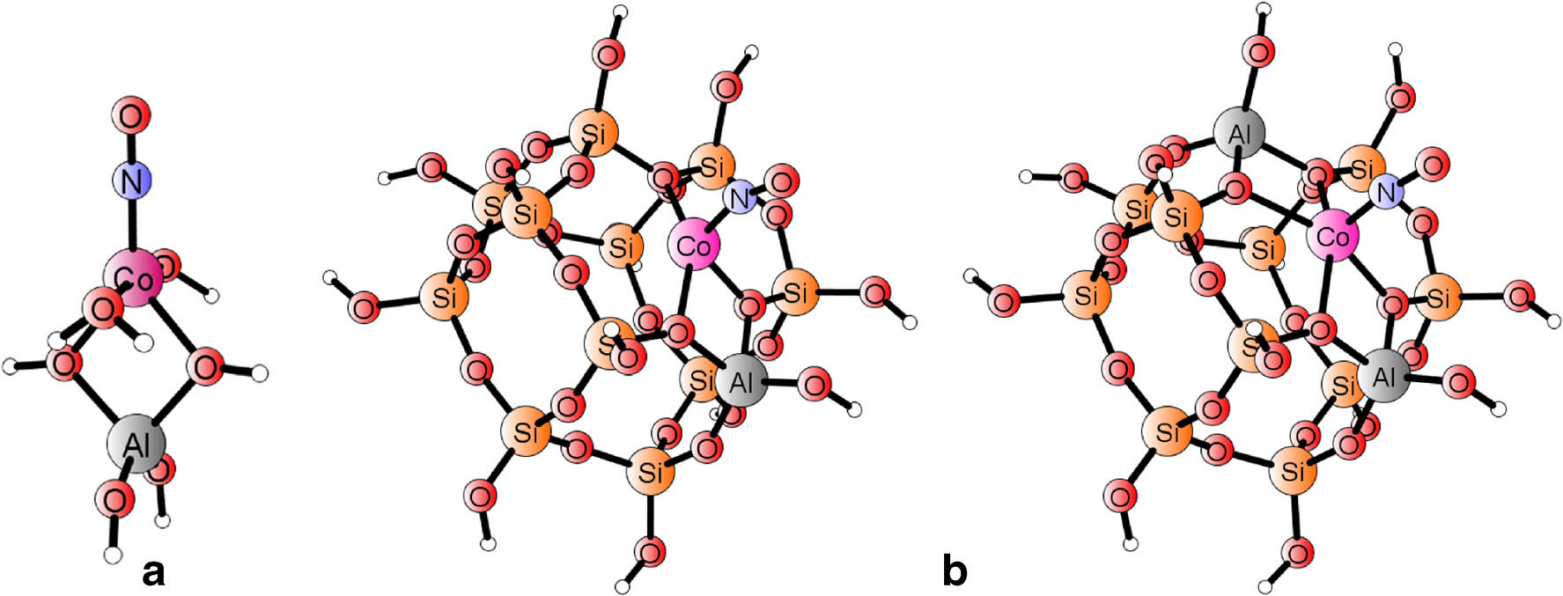
Density functional theory (DFT)-optimized geometries for models of Co(II)–NO adducts (triplet spin states), representing NO adducts for Co(II) site with NO in a 6-member ring. **a** Small cluster [T1(H_2_O)_2_–Co–NO]^+^ . **b** Extended [T12]_1Al_ or [T12]_2Al_ clusters

**Fig 2:**
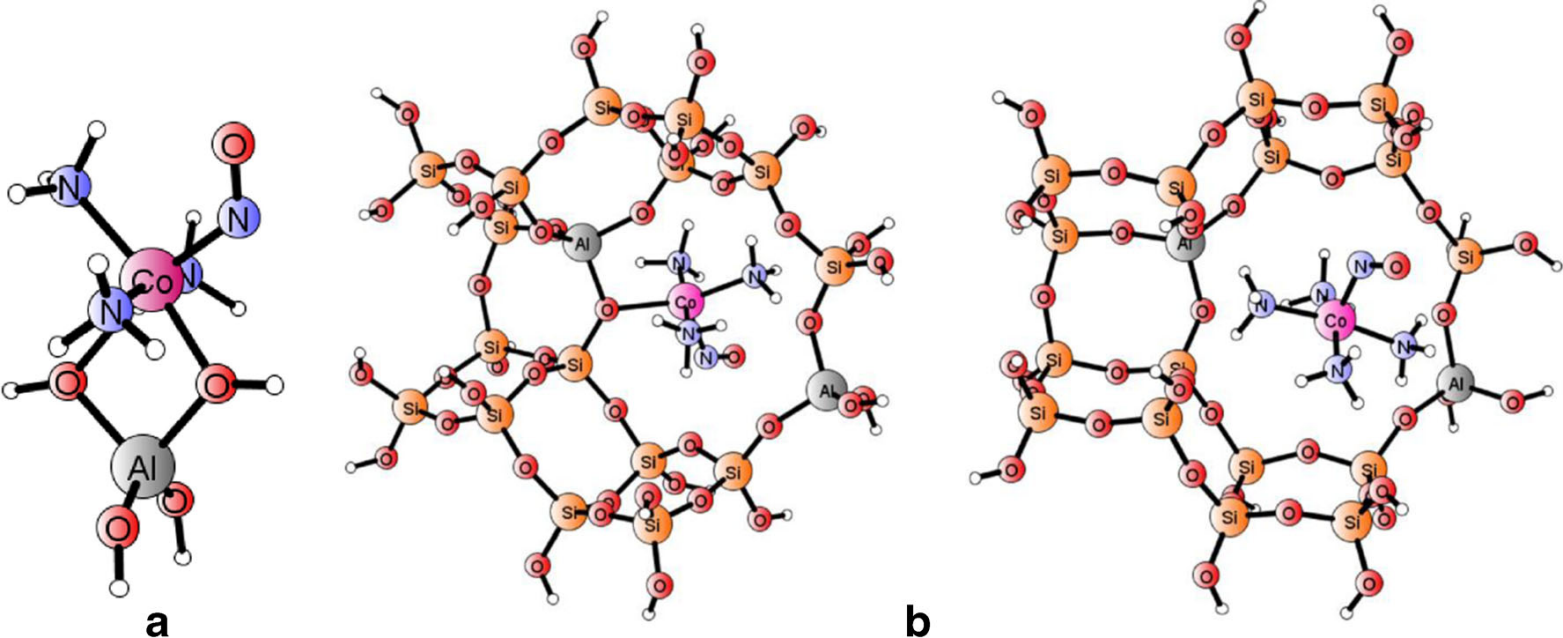
Optimized geometries for models of Co(II)(NH_3_)_n_–NO adducts (singlet spin state), representing adducts of Co(II) site with NO and 3 or 4 ammonia co-ligands, respectively, in a 8-member ring. **a** Small cluster [T1–Co(NH_3_)_3_–NO]^+^. **b** Extended [T18]_2Al_ clusters

Geometry and electronic structure optimization cycles were performed by Turbomole software [27], using the UDFT:BP86 method and the def2-TZVP basis set. NO vibrational frequencies were derived from harmonic approximation (diagonalization of full Hessian for appropriate stable structures). Two types of cluster models were involved; full rationalization of the procedure for small cluster construction has been described in more detail in our former papers [22, 23] and will be recalled here only briefly. The small model for a native Co(II) center in a general zeolite was based on a single-Al tetrahedron (T1), with the Co^II^ cation bound to two T1 oxygens, expressed by the formula [Al(OH)_4_(H_2_O)_2_Co]^+^ (two water molecules were added to saturate two dangling cobalt valences, crudely mimicking framework oxygens). Within this simplistic model, the initial stage of ammonia adsorption was described by the exchange of two water molecules by two ammonia ligands and, in a consecutive step, the third ammonia ligand was added to this adduct. The pentaamminecobalt(II) complex served to model the fully ammonia-saturated system, which was assumed completely unbound to any framework oxygens. Nitric oxide was bonded to the cobalt for all models, and the geometry was optimized into two spin states (singlet and triplet) that were found to be close in energy although showing distinct geometries. Figures 1a and 2a illustrate examples of optimized simplistic models for complexes mimicking the native Co(II)-NO site and the site with three NH_3_ ligands co-adsorbed on cobalt (in triplet and singlet ground spin states, respectively), merely to illustrate our arguments. All other studied T1-based models were discussed extensively in our recent paper [23] and serve here as a reference to discuss new findings.

The extended clusters, based on optimized periodic model of CHA zeolite hosting the relevant adducts (cf. next paragraphs), were used to verify reliability of the simplistic, T1-based models in reproducing major structural features and stretching vibrational frequencies of NO ligand bound to {T1Co(NH_3_)_n_} adducts in a zeolite framework. Three cases are discussed here: (i) the {[Co(II)(NH_3_)_2_]–NO} adduct bound by T12 cluster mimicking a six-membered ring in the CHA chamber (with one or two Al substitutions), and two T18 clusters, modeling the adducts {[Co(II)(NH_3_)_3_]–NO} (ii) or {[Co(II)(NH_3_)_4_]–NO} (iii), both forms found in MD simulations (vide infra) stably bound in the vicinity of an eight-membered ring in the CHA chamber. Optimized geometries of T12 clusters with 1Al or 2Al substitutions and bound Co–NO core are shown in Fig. 1b). The structures of T18 clusters hosting {[Co(II)(NH_3_)_3_]–NO} and {[Co(II)(NH_3_)_4_]–NO} adducts are shown in Fig. 2b).

The two T12 models, +1 charged with 1Al or neutral with 2Al substitutions, were introduced to test the most doubtful assumptions behind a single-Al, T1 model hosting the Co^II^-NO adduct: an unbalanced +1 charge and coarse saturating of two dangling cobalt valences by water molecules. Obviously, reproduction of the adduct geometry is not perfect; nevertheless, fourfold cobalt coordination and nearly linear geometry of the Co–N–O fragment found for realistic neutral T12 cluster with 2Al substitutions (assuming the sequence of Al–(O–Si)_2_–O–Al, after [13, 15]) seems reasonably reproduced by the simplistic model. Other properties of the [T12]_2Al_ model are also fairly well reproduced by the T1 model as discussed in Table 1 in the Results section .

**Table 1:** Shares (in %) of {Co^II^–NO^0^}, {Co^I^–NO^+^} and {Co^III^–NO^-^} resonance structures in total complete active space self consistent field (CASSCF) wavefunction for T1-based models of [Co(II)–NO] adducts with none, two or three ammonia co-ligands, together with measured and calculated shifts of the NO frequency (with respect to free NO (1875 and 1884 cm^−1^ measured and calculated [23])

Model, spin state	∆*ν*_N-O_ (cm^−1^)		Share in CAS function (%)		
	IR experiment estimate	Cluster DFT	NO^0^	NO^+^	NO^−^
[T1Co–NO]^+^(H_2_O)_2_ T	-7	+74	73.3	18.2	8.2
[T1Co–NO]^+^(NH_3_)_2_ S	-	+6	68.1	16.0	15.4
[T1Co–NO]^+^(NH_3_)_2_ T	-	-58	73.4	12.4	13.8
[T1Co–NO]^+^(NH_3_)_3_ S	-250	-226	68.0	6.1	25.1
[T1Co–NO]^+^(NH_3_)_3_ T	-109	-80	72.2	10.9	16.4
[Co–NO]^2+^(NH_3_)_5_ S	-207	-166	75.4	7.5	16.6

Visual inspection of both small and extended models for {Co(II)(NH_3_)_3_–NO} adducts in a zeolite already revealed the close similarity of the core Co–N–O structures based on T1 and [T18]_2Al_ models. The sequence of Al–(O–Si)_3_–O–Al has been assumed for eight-membered ring in accordance with the high-silica nature of our periodic model (Si/Al ≈ 11); the position of the second Al node seems still less relevant in this case as the adduct with three NH_3_ apparently losses one connection to framework oxygens, and remains in the vicinity of a single AlO_4_ tetrahedron while the binding of four ammonia ligands and NO to Co(II) center breaks covalent bonding of the [Co(II)(NH_3_)_4_]–NO] core with the framework. The issue of the impact of additional ligation of the Co(II) center on the dependence of its siting upon Al distribution will be discussed in more detail in connection to dynamic simulation. Full comparison of most important geometric and spectral features given by the {[T1Co(NH_3_)_3_]^+^–NO} and {[T18Co(NH_3_)_3_]–NO} clusters is given in Table 2 in the Results section.

**Table 2:** The comparison of structural and electronic properties calculated for T1-based and [T12]_1Al_ or [T12]_2Al_ models of the native Co(II)–NO site in the triplet ground state

	[T1(H_2_O)_2_]	[T12]_1Al_	[T12]_2Al_
Angle (°)			
Co-N-O	180	160.6	161.6
Bond lengths (Å)			
Co-NO	1.69	1.70	1.70
N-O	1.14	1.14	1.15
Co-O	1.97; 1.97 2.19*; 2.19*	1.95; 2.05 2.19; 2.76	2.05; 2.23 2.08; 2.19
Electronic properties			
Q_Co_	+0.55	+0.56	+0.54
Q_NO_	+0.22	+0.22	+0.18
ρ^S^_Co_	2.19	2.03	2.09
ρ^S^_NO_	0.43	0.35	0.43
NO frequency (cm^−1^)			
∆v_NO_^C^	+74	+44	+28

^*^O_H2O_ in T1, equivalent to O_Al_ in T12 models

#### CASSCF calculations of electron density distribution

Single point calculations for wavefunction analysis and spin state energetics were performed by means of Molcas 7.6 software [28] for DFT:BP86 optimized geometries of relevant T1-based models. Details on MOLCAS calculation protocol are given in [23]. Complete active space self consistent field (CASSCF) wavefunction yields (by default) delocalized natural molecular orbitals. Thus the construction of modified natural orbitals, localized within the active space and serving to define configurations built of π_NO_, π_NO_* or d_Co_ like orbitals (used to extract VB-like resonance structures) calls for special procedure. The modified procedure (based on Pipek-Mezey algorithm) has already been proposed and successfully tested in our former papers [23, 29, 32]. By expressing the multiconfigurational CASSCF wavefunction in terms of (partly) localized active orbitals, it is possible to read the wave function in terms of resonance structures with a definite number of electrons assigned to the π_NO_ and π_NO_* orbitals (on NO fragment), and the d_Co_ orbitals (on Co(II) fragment). In this way, all components of the wavefunction may be ascribed either to {Co^II^–NO^0^}, to {Co^III^–NO^-^} or to {Co^I^–NO^+^} VB-like electronic structures; the sum of weights of the appropriate configurations yields total cumulative importance of the respective electronic form for a given system.

### Periodic CHA models and born-Oppenheimer MD

VASP code [30] was used to perform periodic DFT calculations. Kohn-Sham equations were solved in the plane-wave basis set with the projector-augmented-wave (PAW) method of Blochl, as adapted by Kresse and Joubert. The exchange-correlation functional was represented by the Perdew-Burke-Ernzerhof (PBE) approximation. Brillouin-zone sampling was restricted to the Γ point. The plane-wave cutoff of 300 and 400 eV was used in MD simulations and geometry optimizations, respectively. Spin polarization mode was switched on for all calculations. In order to investigate the close lying spin states, we fixed the magnetic moment. The criterion for electronic self-consistency cycle was set to 10^−8^ eV/cell for geometry optimizations and 10^−4^ eV/cell for MD simulations. Gaussian smearing (with smearing parameter of 0.01 eV) was applied. Conjugate gradient algorithm was employed for structural optimizations at constant volume with fixed shape and size of the unit cell. In the relaxed structures, all forces acting on atoms were smaller than 0.03 eV/A.

Our model for periodic calculations has been based on the chabasite structure, assuming a doubled, 24-T unit cell with 2 Al substitutions, yielding Si/Al equal to 11. It does not mimic actual IR-studied systems or perfect distribution of Al sites but allows for reasonable size of the model. Restricting the size of the model and lowering computational protocol requirements were necessary to reach calculation feasibility and execute ab initio Born-Oppenheimer (BO) dynamics for Co^II^ complexes with NO, where many forms of importance, as well geometrical as electronic could be expected [22, 23]. We have applied BO dynamics implemented in VASP since we regarded it better suitable for our goal than either Carr-Parrinello MD (found effective for Cu–NO systems in SSZ-13 [16]), which might overlook selected states in the case of cobalt, or classical MD simulations (used recently to describe mobility of ammonia during the NH_3_-SCR process [31]. We are perfectly aware that, within our protocol and limited time, only suggestions or preliminary conclusions could be reached. They are backed, however, by our long experience with the system of interest. BO MD simulations were performed in canonical NVT ensemble where the temperature was controlled by a Nose-Hoover thermostat. The Verlet algorithm was used to integrate equations of motion for atoms, an integration step of 1 fs was applied, and the length of trajectories was 50 ps. The mass of hydrogen was set to 3 amu. The simulation temperature was set to 300 K. Langevin dynamics was used for the free molecule of nitrogen oxide. In order to identify the optimal structure and energy of non-modified cobalt site in six-ring, 50 structures randomly selected from the corresponding MD trajectory were used for static geometry optimization described below.

#### Model optimization

Lattice vectors for basal periodic model were derived from experimental data determined for highly siliceous form of chabasite (R3m, a = 9.291 Å, α = 93,92 deg.). Extended supercell defined by translational vectors a = 12.682 Å, b = 13.581 Å, c = 9.291 Å, α = 90.00°, β = 95.74°, γ = 90.00° and containing 24 tetrahedral units was used to avoid the undesired interactions between the reactive domain and its periodically repeated images (the distance Co–Co kept larger than 5 Å). Two Si atoms were substituted by Al atoms to create negative charge counter-balancing the positive charge of Co^II^ cation. We took under consideration the sequence of Al–(O–Si)_2_–O–Al identified in recent experimental study by Dedecek et al. [14, 15] to be the most likely Al sequence in a six-membered ring in zeolites with high Si/Al ratio (Si/Al ≈ 11 in our model). In the eight-membered ring, the sequence Al–(O–Si)_3_–O–Al has consistently emerged; this deficiency of close Al pairs in 8MR fragments does not contradict strong suggestion of Dedecek [13–15] that Al pairing should be crucial for the binding of Co^II^ by the framework as only after binding more than two ammonia co-ligands does the Co(II)-NO adduct partly lose contact with the framework and migrate from the preferred site in 6MR to more open space in 8MR vicinity (*vide infra*). Indeed, the Co^II^ –NO adduct in its triplet ground spin state, initially placed in a six-membered ring (6MR) remained stably bound when the geometry was relaxed after the MD run. Next, the initial models of ammonia ligated Co(II)–NO forms were constructed by binding consecutive NH_3_ ligands (with* n* increasing from 1 to 3) and the search for the stable structures was repeated for singlet and triplet spin states.

**Fig 3:**
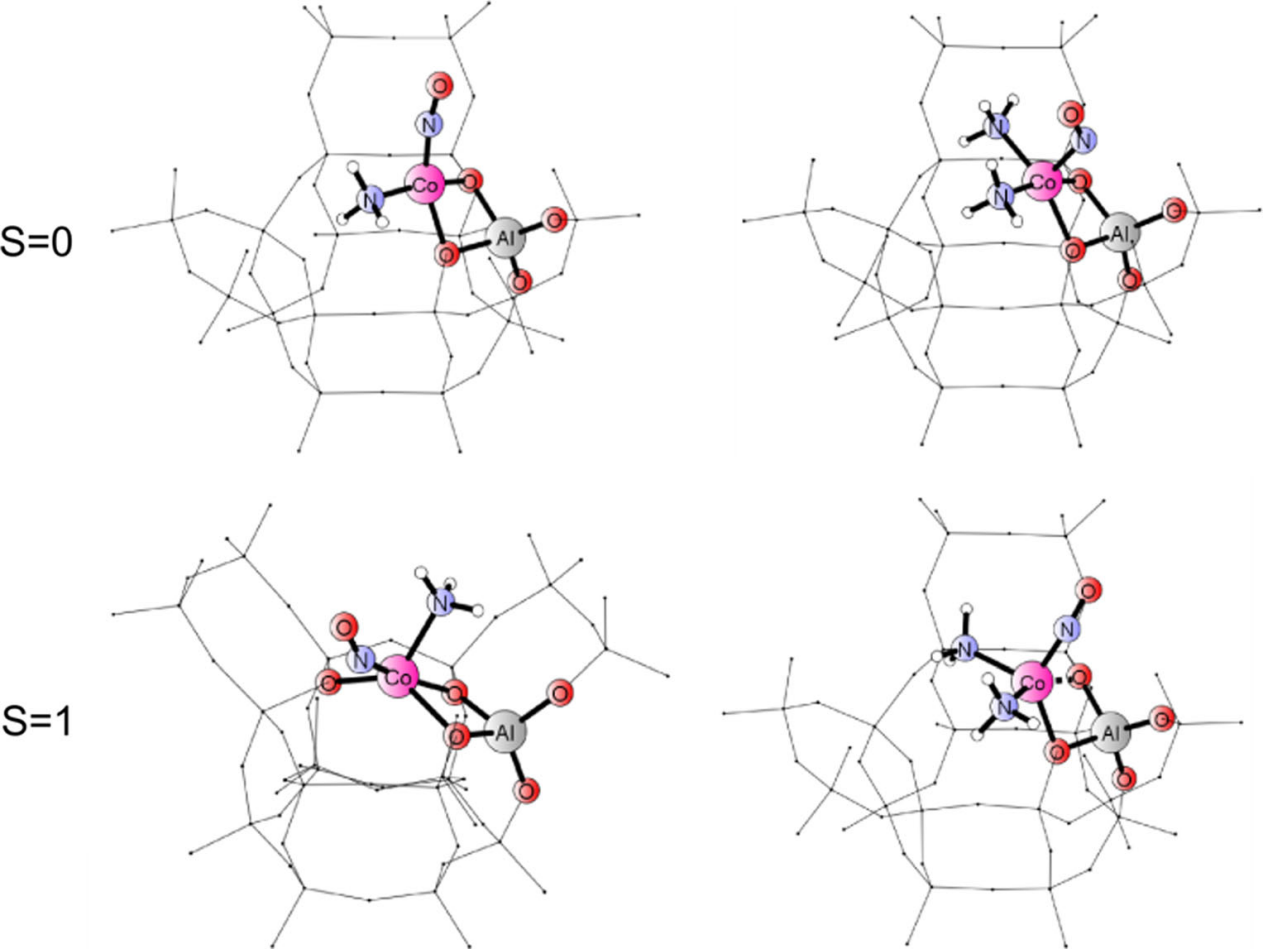
Minimum energy structures for Co(II) adducts {(NH_3_)_x_–Co(II)–NO}, with x = 1 (left panel) or 2 (right panel) in a six-membered ring for singlet and triplet spin states; atoms corresponding to primitive cluster models depicted by balls and sticks

**Fig. 4:**
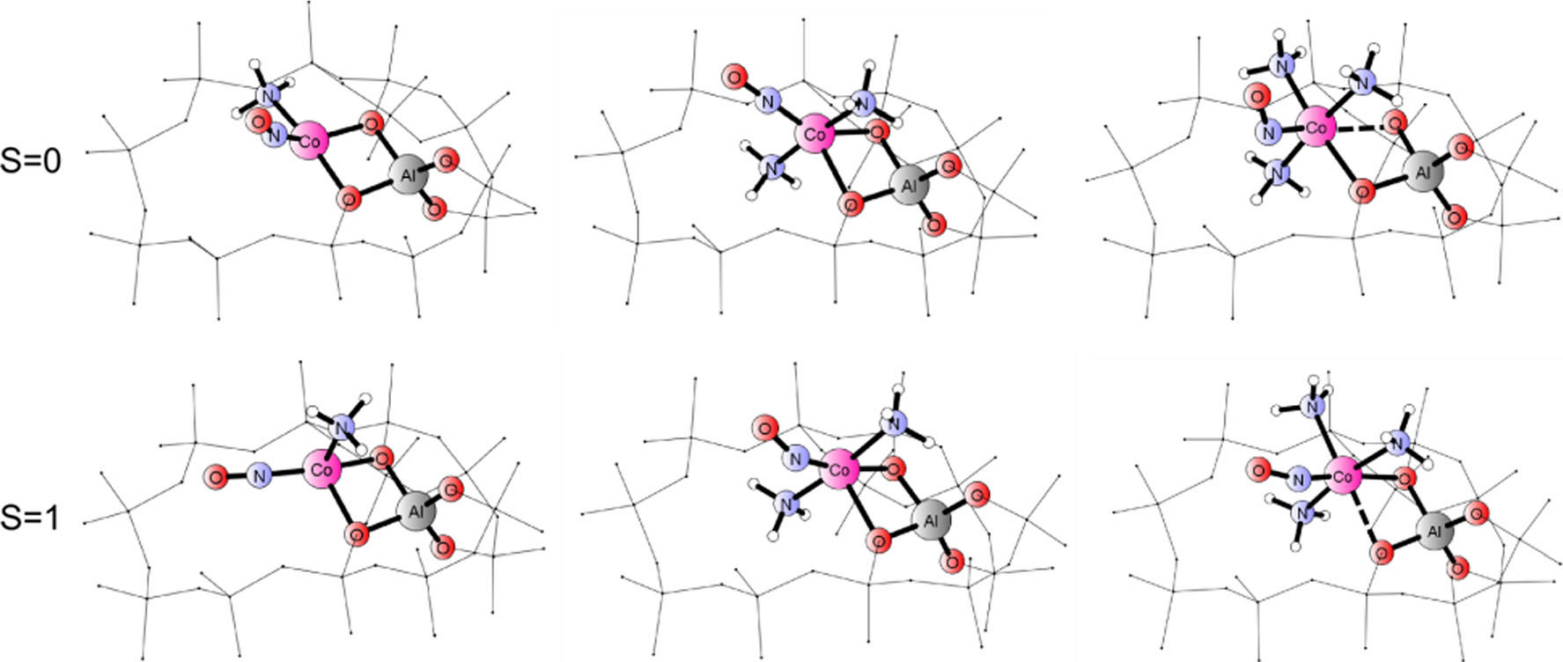
Minimum energy structures for Co(II) adducts {(NH_3_)_x_–Co(II)–NO}, with x = 1, 2 or 3 in eight-member ring for singlet and triplet spin states; atoms corresponding to primitive cluster models depicted by balls and sticks

Figure 3 shows the optimized structures for framework fragments comprising adducts with one (left panel) or two (right panel) ammonia ligands in the two spin states (atoms corresponding to those in primitive cluster models depicted by balls and sticks). Minimum energy structures suggest that in the triplet (ground state for the native Co(II)–NO adduct) the addition of consecutive ammonia co-ligands gradually breaks the bonding to framework oxygens in the six-ring (keeping five-fold coordination of Co–NO). The singlet adducts are generally weaker bound to the framework: already the first NH_3_ breaks one cobalt bond to oxygen. The third ammonia molecule did not bind stably in neither spin state for the adducts remaining in the area of the six-membered ring. Since the structures with two NH_3_ co-ligands showed the tendency to shift towards the eight-membered ring, we constructed new initial models by positioning complexes with 1, 2 or 3 ammonia co-ligands above the next-neighbor, eight-membered ring and the optimization procedure was repeated. Figure 4 shows the minimum energy structures for framework fragments comprising adducts with one, two or three ammonia ligands in the vicinity of eight-membered ring (in the two spin states).

## Results and discussion

### Resume of small-cluster calculations

Full description of the outcome of DFT calculations, based on small clusters (T1) taken as basal models of Co(II) active sites, carried out to obtain properties of Co(II)–NO adducts in ammonia-pretreated zeolites, has already been given in our former papers [22, 23]. Here, we briefly summarize our findings, focusing on the electronic background of the N–O bond activation by cobalt active sites. We have qualitatively reproduced major features of the IR spectra (in NO stretching frequency region) taken for MOR, BEA and FER zeolites in two distinct experimental conditions, namely after intermediate and full saturation of the zeolite framework with ammonia prior to NO sorption. The doses were calibrated to correspond to the average number of either 3 or 4.5 NH_3_ molecules bound per Co(II) site (after outgassing), respectively. Straightforward interpretation occurring after inspection of FTIR spectra obtained for the two types of samples suggested to ascribe the higher frequency peaks, corresponding to the NO stretch red-shifted by ca. 100–130 cm^−1^ (with respect to free NO), to the adducts of the type {[Co(II)(NH_3_)_3_]–NO}. Still lower frequency range (red-shifted by 190–200 cm^−1^, dependent on the zeolite type) was blindly assigned to the NO stretch in a zeolite hosting {[Co(II)(NH_3_)_5_]-NO} forms immobilized in cages. Our calculations of NO stretching frequencies for small cluster models suggested that it were the adduct with three ammonia ligands co-bound to cobalt (in the singlet spin state, being still linked to the negative framework) that produced the highest red-shift of the NO frequency of over 200 cm^−1^ and yielded the low-frequency range of the experimental spectrum. On the other hand, the singlet adduct {[Co(II)(NH_3_)_5_]-NO}, no more capable of forming bonds with cluster oxygens representing electron-rich framework, showed smaller red-shift of the NO stretch (ca. −160 cm^−1^) and was pointed responsible for the intermediate-frequency region of the experimental spectrum. These findings led to tentative re-interpretation of the FTIR spectra.

However, the most appealing part of our last paper [23] concerned the enriched rationalization of the shift of NO stretching frequency upon interaction with the active site. We have shown that small cluster models appeared qualitatively well-working mimics of the actual active site; their huge advantage came from their modest size and thus applicability of the correlated wavefunction methodology (CASSCF). As already stated before, Co(II)–NO adducts belong to the class of non-innocent systems, where the reliability of DFT may be questioned, which calls for high-level quantum chemical methods for exact description of electronic structure and electron density. In particular, the distribution of the electron density within the fragments forming the non-innocent complex calls for special care as noted by us in former papers [23, 29, 32]. In the recent papers on Co(II)–NO and Fe–NO complexes [23, 32], we have shown that the accurate and reliable method to extract electron density redistribution was the Valence-Bond (VB)-like expansion of the CASSCF wave function in terms of resonance structures, obtained by expressing the multiconfigurational CASSCF wave function in terms of localized active orbitals. By this procedure, one can read the wave function as composed of resonance structures with a definite number of electrons assigned to the π_NO_ and π_NO_* orbitals (NO fragment), and the d_Co_ orbitals (Co(II) fragment). We successfully followed this procedure in our last work, and obtained valuable insights into the mechanism of the Co–NO bond formation, as briefly summarized in the following paragraphs.

Table 1 summarizes (after [23]) the most appealing results extracted from correlated wavefunction analysis: the relative shares of relevant valence structures in the total CASSCF function for simplistic, T1-based models of the native Co(II) site with adsorbed NO molecule (in the triplet state), and of the cobalt site hosting 2 or 3 ammonia co-ligands in singlet or triplet state, and the complex with five ammonia co-ligands in its experimentally inferred singlet ground state. Full discussion of these data has already been given in our recent paper; here, we collectively summarize the main findings, with the stress put on far-reaching conclusions. It is clearly visible that the relative weights of the major resonance structures ({Co^II^–NO^0^}, {Co^III^–NO^-^} or {Co^I^–NO^+^}) align very well with calculated ∆*ν*_N–O_ shifts. Apparently, the adsorption complex Co(II)–NO with three NH_3_ ligands (still linked to T1 oxygens) presents both the biggest red shift of the NO stretch and the largest contribution of the {Co^III^–NO^-^} valence structure to the wavefunction. On the other hand, the model corresponding to the NO ligand bonded to the native Co(II) site shows both the largest contribution of the valence structure corresponding to the {Co^I^–NO^+^} form and the largest blue shift of the NO stretching frequency.

These results confirm the assumption (recalled frequently in coordination chemistry and catalysis) that the activation of the NO molecule upon the interaction with transition metal sites relies strongly on the site donor properties and follows closely the Chatt-Duncanson proposal for the backdonation as a major driving force for NO activation by transition metal centers. It seems thus well grounded to propose the relative weights of the both {Co^III^–NO^-^} and {Co^I^–NO^+^} resonance structures in the total wavefunction as semi-quantitative descriptors of the importance of donation and backdonation processes for a formation of the coordinative bond, respectively. Indeed, the comparison of the singlet pentaamminenitrozocobalt(II) complex with the triplet adduct binding three ammonia ligands shows that while both present comparably large shares of the NO^−^ form, they substantially differ in the share of {Co^III^–NO^+^} component. The increased value of the latter for the triplet adduct goes in line with the much smaller red-shift of *ν*_N-O_ in this case. Therefore these two electron transfers are apparently operating together, and it is the balance between them that decides upon final activation of the NO molecule.

### Extended cluster studies

Prompted by important novel clues stemming from our previous papers and summarized in the former paragraph, we have extended cluster models (by following periodic framework structures, vide supra) in order to check the validity of inferred general conclusions for actual zeolite systems and better approach experimental spectra. The initial periodic model for each case was prepared by positioning the appropriate adduct in a selected region of a periodic framework of CHA zeolite (prepared as described in the Methods section). After careful analysis of the behavior of the adducts with consecutive ammonia ligands attached to the Co–NO core (see also next section), we selected the T12 cluster as a good representative of the framework hosting the native Co(II)–NO and adducts with 1 or 2 NH_3_ co-ligands, found stable in the vicinity of the 6MR ring with the Al–(O–Si)_2_–O–Al sequence, cf. Fig. 1 (in accord with prediction by Sobalik et al. [14, 15]). In addition, we constructed a similar model for the native cobalt site with a single Al substitution to probe the most questioned deficiency of the simplistic, T1-based model, namely, the positive charge of a model due to a single Al availability for a divalent cobalt cation. The T18 cluster model with two Al substitutions was used to represented the adducts comprising two and more ammonia ligands (cf. Fig. 2).

Table 2 compares most important geometric parameters for simplistic, T1-based model and positively charged [T12]_1Al_ or neutral [T12]_2Al_ models of the native site hosting the Co(II)–NO adduct in a six-ring. In addition, atomic charges, NO spin density and the shift of the NO stretching frequency are compared. By no means did we expect quantitative agreement, especially with respect to “soft” parameters like the Co–N–O angle and the NO stretching frequency. Nevertheless, it is clearly visible that the positively charged (T12)_1Al_ model reproduces most properties of the adduct obtained from the more realistic, neutral (T12)_2Al_ model surprisingly well. In consequence, the still less reliable simplistic [T1(H_2_O)_2_Co]^+^–NO model behaves consistently well, which significantly reduces methodological concerns regarding our former studies. In addition, our results seem to indicate that the compensation of the surplus positive charge imposed by a divalent cation could be also effectively neutralized in a real zeolite by long-range interaction with the distant negative charge.

Encouraged by positive results of the most demanding test for the native cobalt site with bound NO ligand, we set the calculations for two-ammonia and three-ammonia ligated cobalt Co–NO core. Table 3 compares most important geometric parameters and the shift of the NO stretching frequency for simplistic, T1-based and extended [T18]_2Al_-based models of the site hosting Co(II)-NO adduct with three ammonia co-ligands in an eight-membered ring. Again, the simplistic [T1Co(NH_3_]_3_]^+^–NO model reproduces very well those given by the realistic [T12]_2Al_ model, which further confirms the reliability of our conclusions concerning the influence of ammonia co-ligands on the activation of NO ligand by cobalt site in a zeolite.

**Table 3:** Selected geometric and spectral features given by {[T1Co(NH_3_)_3_]^+^–NO} and {[T18Co(NH_3_)_3_]–NO} cluster models of {Co(II)(NH_3_)_3_–NO} adducts (in singlet ground state)

	[T1Co(NH_3_)_3_]^+^–NO	[T18Co(NH_3_)_3_]_2Al_–NO
Angle (°)		
Co–N–O	122.4	122.8
Bond lengths (Å)		
CoNO	1.79	1.78
N–O	1.19	1.18
Co–O	1.94 2.20	2.08 2.64
Co–NH_3_	2.00 2.00 2.00	1.99 2.00 1.96
NO frequency (cm^−1^)		
Δν_NO_	−226	−217

### Periodic MD simulations

The promising results presented above prompted us to extend our computational protocol to periodic framework structures. Here, we present the results of extended studies within the realistic, periodic model of a chabasite zeolite to further confirm the validity of our conclusions for actual systems. The minimum energy geometry after structure optimization was selected as the initial structure for each of the studied systems. All systems were next subjected to MD protocol in two spin states, starting from the appropriate adduct (with structure predicted by cluster calculations) placed in the vicinity of either six- or eight-membered ring in the minimized periodic framework. Tables S1 and S2 in Electronic Supplementary Material (ESI) list important parameters characterizing consecutive ammonia co-ligated systems in the singlet and triplet spin states: geometrical parameters of the optimized structures, for up to two-ammonia adducts over the six-ring (Table S1), and for one- to three-ammonia adducts over the eight-membered ring (Table S2), respectively. For distances, the averages over MD run are also given for each trajectory, together with the cobalt distance from the framework plane set by 3 T atoms, its coordination number and anharmonic NO stretching frequency. Anharmonic stretching frequencies of the NO ligand were extracted from 50 ps MD by means of velocity autocorrelation function (restricted to N and O atoms) according to the protocol based on VASP code [30, 33] for all experimentally-oriented systems of interest. All relevant power spectra of velocity autocorrelation function are shown in Figs S1 – S8 in the ESI. We assumed a 1 fs time step to be sufficient as we did not aim for high frequency vibrations involving hydrogens. For ammonia-deficient conditions, optimized geometries of {Co^2+^–NO–(NH_3_)_2_} and {Co^2+^–NO–(NH_3_)_3_} systems served as initial structures for MD run while for the {Co^2+^–NO–(NH_3_)_3_ + 4NH_3_} model (simulating the system upon ammonia surplus) modified protocol has been applied (vide infra).

Table 4 summarizes the results of analysis of Co(II)-NO adducts comprising either no other ligand or two and three ammonia co-ligands bonded to the cobalt center in a periodic CHA framework. The table lists the most important structural properties (extracted from the 50 ps MD trajectory), characterizing the behavior of each adduct embedded in an actual zeolite framework. Apparently, the average cobalt coordination number to framework oxygens (LK) and the distance between the Co cation and the plane defined by the three T atoms (PD) depends on the number of ammonia co-ligands as well as on the spin state of the adduct. The general rule may be observed that the cobalt coordination number to framework oxygens decreases, and the distance from the surface increases with increasing number of bound NH_3_ ligands. This could be intuitively foreseen but a new issue emerges from the spin dependence of these parameters. In general, the singlet spin state prompts a tighter binding to the framework (apart from the native Co(II)–NO site) while the triplet weakens the interaction. We shall comment on this issue after discussing the results of our MD simulations. For the complex with three NH_3_ ligands, the rule seemed not to hold; however, more sophisticated modeling of the system upon ammonia surplus condition finally confirmed this suggestion (vide infra).

**Table 4:** Results of MD simulations for model systems composed of a periodic CHA framework and the [Co(II)–NO] adducts comprising none, two or three ammonia co-ligands bonded to the cobalt center: average cobalt coordination number to framework oxygens (LK) and the distance from the ring plane (PD)

Model	Spin state	6MR		8MR	
		LK	PD	LK	PD
Co^II^-NO	Singlet	2.01	0.75	─	─
	Triplet	1.78	0.95	─	─
Co^II^ -NO-(NH_3_)_2_	Singlet	1.12	1.92	0.99	0.91
	Triplet	0.80	2.29	1.01	0.82
Co^II^ -NO-(NH_3_)_3_	Singlet	─	─	0.78	1.16
	Triplet	─	─	0.73	1.23

Analysis of the simulation trajectories shows that up to two ammonia ligands bind to the cobalt center, initially stably anchored to the framework in the vicinity of a six-membered ring; the adduct with three NH_3_ molecules is not stable in this position (the third ligand dynamically detaches from the cobalt center). However, for the adduct with two ammonia ligands, the positions close to the six-membered and eight-membered rings are energetically comparable. Since the ground state energies for optimal geometry of this complex in the two spin states are also very close, we suggest that, upon ligation by ammonia, cobalt may both change spin state and migrate over the main CHA chamber, enabling binding of the third NH_3_ molecule to the complex in more void space.

**Fig. 5:**
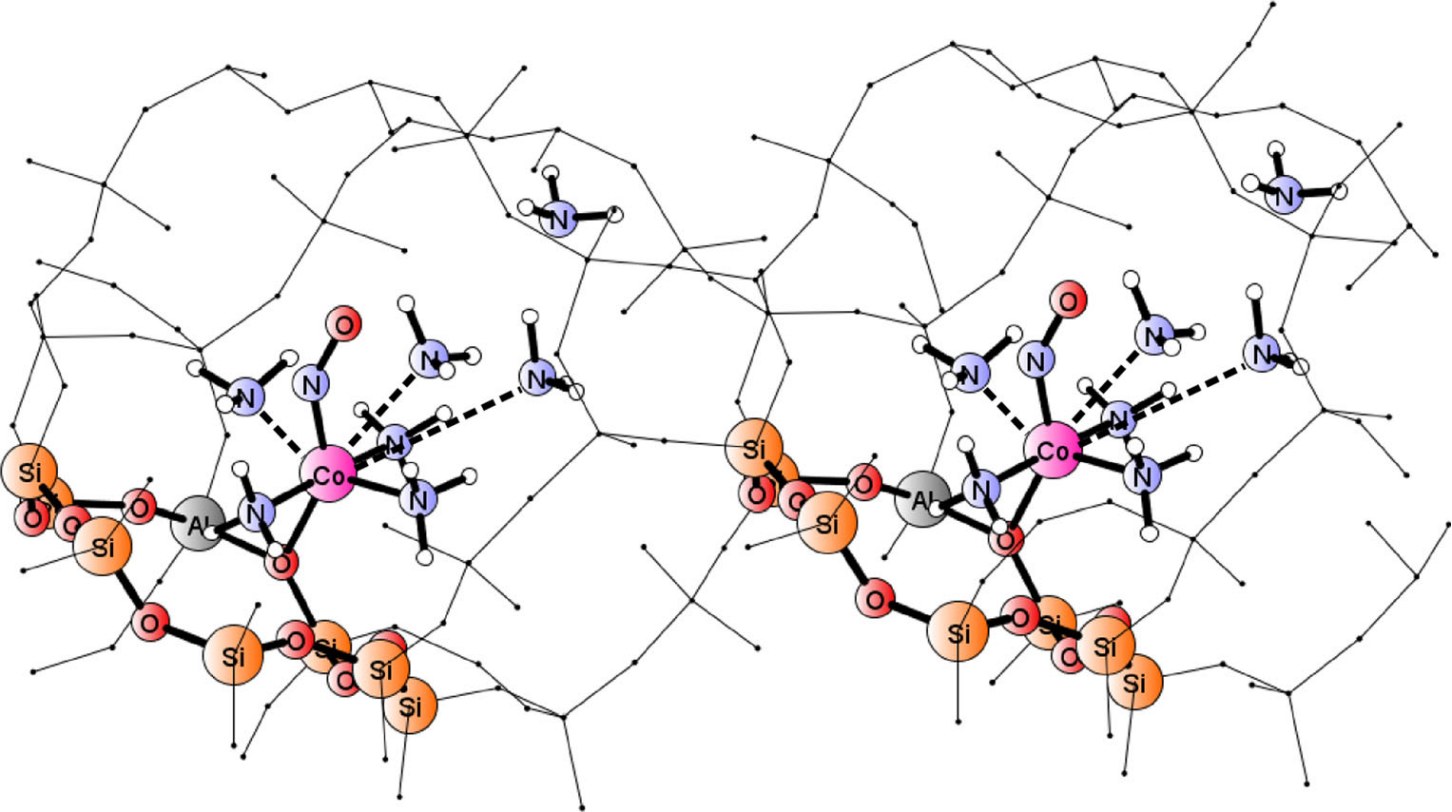
A snapshot from the S1 trajectory showing a fragment of periodic framework with two Co(II)–NO centers, bound to one framework oxygen and strongly coordinating three ammonia ligands, with four others forming the second coordination sphere

**Fig. 6:**
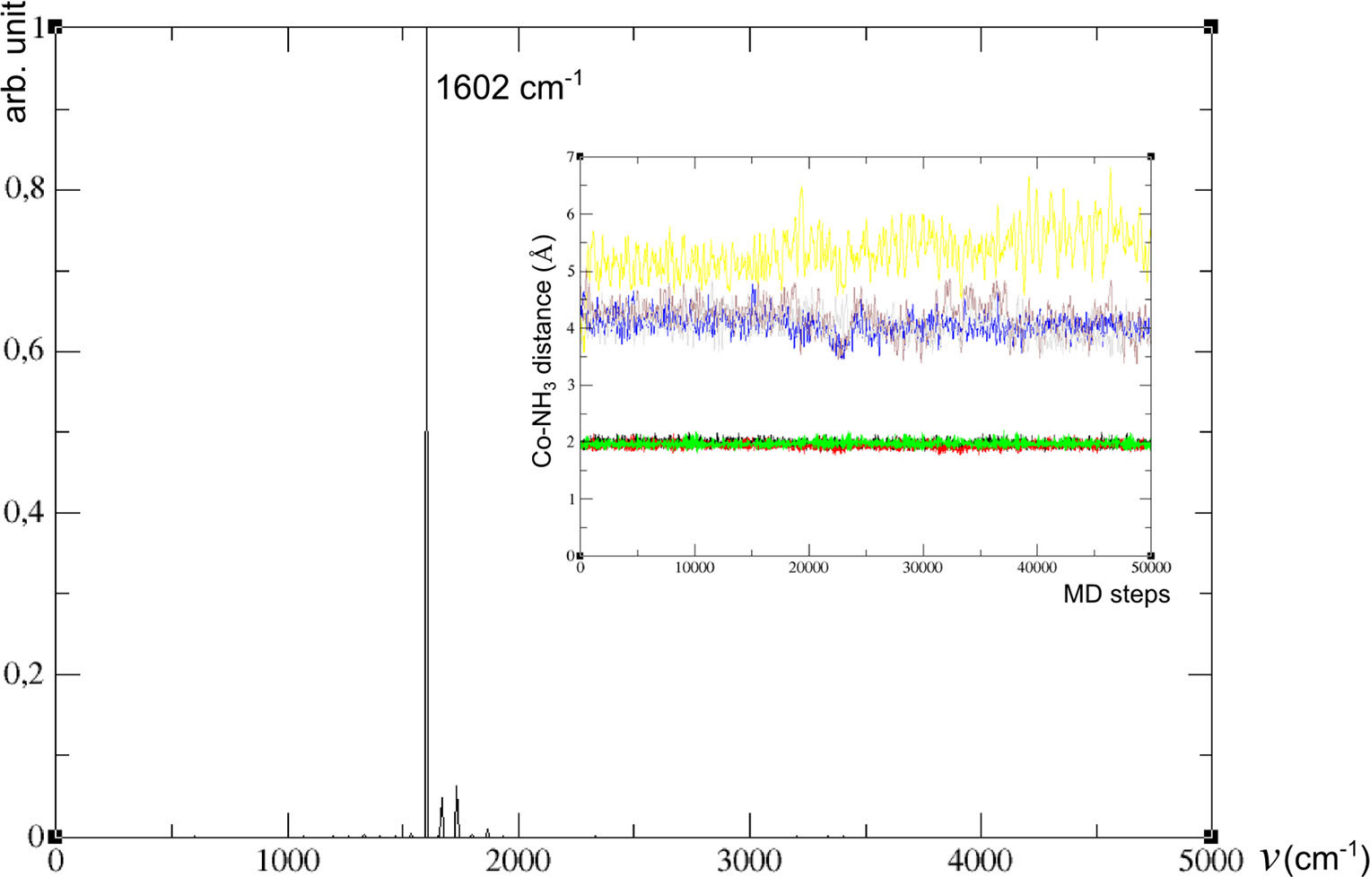
Power spectrum from velocity autocorrelation function filtered with respect to N_NO_ and O_NO_ coordinates for S1-type singlet simulation for the {Co^2+^–NO–(NH_3_)_3_ + 4NH_3_} system; dynamic evolution of Co–NH_3_ distances along MD trajectory shown in the inset (*red*,* green*,* black*,* blue*,* purple*,* grey* and* yellow* mark seven Co–N_NH3_ distances)

**Fig. 7:**
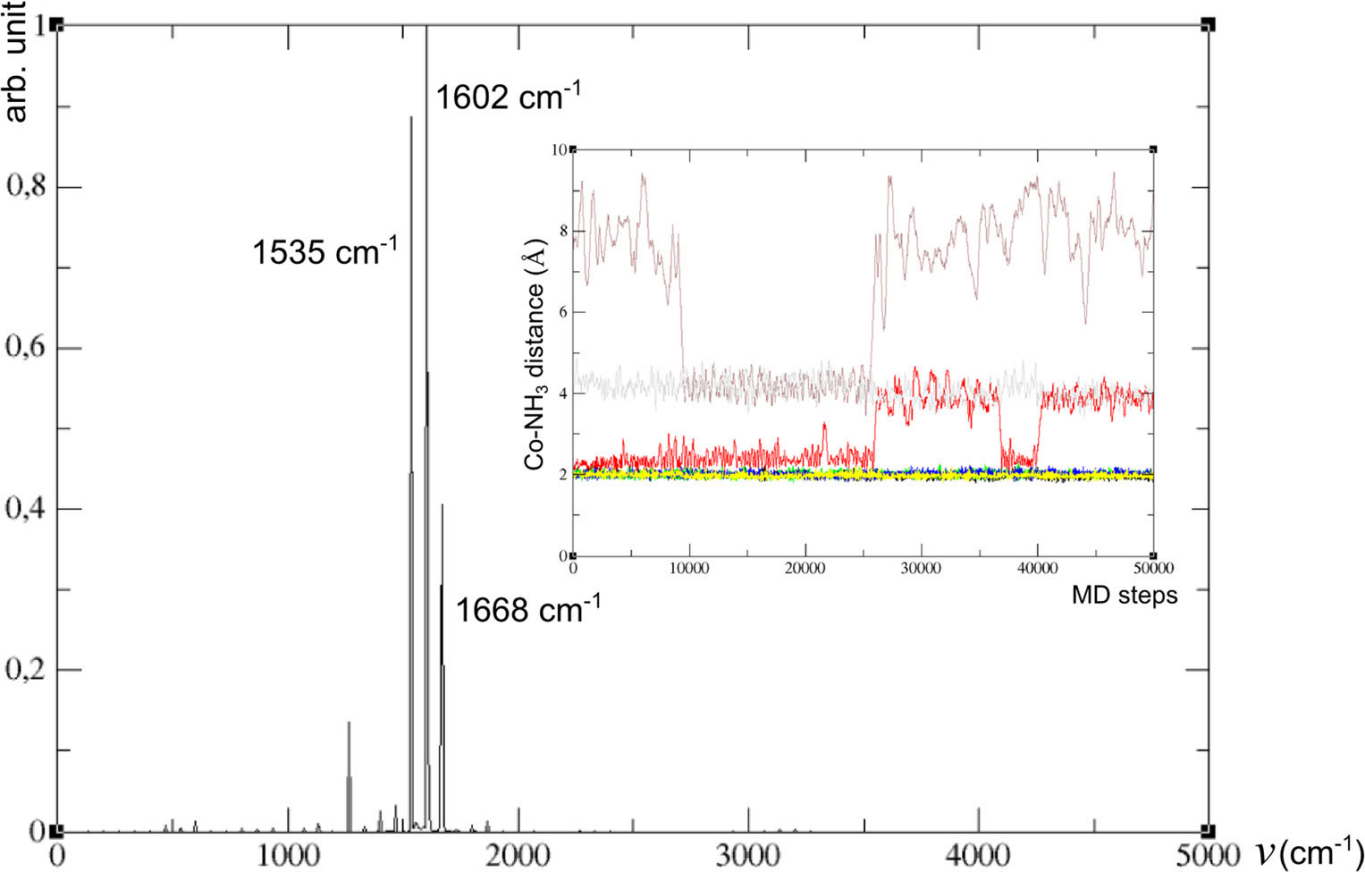
Power spectrum from velocity autocorrelation function filtered with respect to N_NO_ and O_NO_ coordinates for the S2-type singlet simulation for the {Co^2+^–NO–(NH_3_)_3_ + 4NH_3_} system; dynamic evolution of Co-NH_3_ distances along MD trajectory shown in the inset (*red*,* green*,* black*,* blue*,* purple*,* grey* and* yellow* mark seven Co–N_NH3_ distances)

Since the pentaamminenitrozocobalt(II) complex did not stably bind to the framework, a different MD protocol was applied in this case. The dynamic behavior of the fully ammonia-saturated system inside the CHA chamber was simulated by positioning the [Co(II)–NO] adduct comprising three ammonia ligands together with four additional NH_3_ molecules (forming {Co^2+^–NO–(NH_3_)_3_ + 4NH_3_} system) put in the framework (with Co center close to the eight-membered ring). Qualitatively similar trajectories have been obtained from 50 ps MD simulations for the singlet (the ground state for free pentaammine complex) and for the triplet states (see Table 5). The analyses of both trajectories indicated, however, that the system stayed closer to the framework in the case of singlet simulation than for the triplet simulation (LK = 0.55 and PD =1.47 Å for the singlet with respective values of 0.02 and 4.43 Å for the triplet state, see Table 5 and Fig. 5). To further explore the dynamic behavior of the singlet system, we ran another 50 ps simulation starting from initial geometry of the adduct detached from the framework (obtained from triplet simulation) and with the multiplicity set to 1. The modified trajectory (S2) revealed the very different behavior of the system in this case compared to that for initial singlet simulation (S1), with the adduct remaining detached from the framework. The average total energy for the triplet was much higher than those of the singlet states, with the latter two differing by ca. 13 kcal mol^−1^ (not conclusive for the actual ground state in view of intrinsic errors for DFT spin states energetics). The average distances between the cobalt center and all ammonia ligands for two singlet simulation types are given in Table 5. Insets in Figs. 6 and 7 illustrate the evolution of Co–NH_3_ distances along 50 ps trajectory for the two simulation types for singlet state of the {Co^2+^–NO–(NH_3_)_3_ + 4NH_3_} system, respectively.

**Table 5:** Average Co–NH_3_ distances from 50 ps trajectories run for {Co–NO–(NH_3_)_3_ + 4NH_3_} (from two distinct initial conditions for the singlet (S1 and S2) and one for the triplet (T))

Model	Average Co distances to 7 NH_3_ molecules (Å)
S1	1.98 1.94 1.98 4.06 5.35 4.19 4.05
S2	1.98 3.02 2.01 2.02 1.98 6.65 4.11
T	2.02 2.02 2.06 3.83 3.88 4.97 4.44

A relatively simple dynamic behavior of ammonia ligands was observed for the S1-type singlet (cf. Figs. 5 and 6): three ammonia ligands remain stably bonded to the cobalt center throughout the simulation, with the other four NH_3_ molecules dynamically forming the second coordination sphere. Interestingly, the interaction of the Co-centered adduct with the framework is not negligible (LK = 0.55). On the contrary, the S2-type trajectory shows rather intricate character and corresponds to dynamically changeable system, with four ammonia ligands stably bound to cobalt, and the fifth dynamically binding to and departing from the cobalt center (see inset in Fig. 7). The larger average number of ammonia ligands bound to Co(II)–NO core result in a much weaker interaction with the framework (LK = 0.06, see Table 6). The analysis of Co–NH_3_ distances along the triplet (T) trajectory (not shown) is similar to that for the first singlet, although showing somewhat longer average Co–NH_3_ distances: three ammonia ligands remain stably bonded to the cobalt center thorough the simulation, with the other four NH_3_ molecules dynamically forming the second coordination sphere. The other difference lies in negligible bonding of Co-centered adduct to the framework (LK = 0.02).

**Table 6:** Analysis of Born-Oppenheimer (BO) molecular dynamics (MD) trajectories for system corresponding to {Co^2+^–NO–(NH_3_)_3_ + 4NH_3_} embedded in CHA framework: NO frequency shift (∆v_NO_^P^ – from MD power spectrum, with respect to 1830 cm^-1^ for free NO), coordination number of Co to framework oxygens LK, average distance of Co from the ring plane PD (Ǻ)

State	∆*ν*_NO_^P^	LK	PD	Major form
S1	−228^a^	0.55	1.47	Co-NO-(NH_3_)_3_ + 4NH_3,gas_ Weakly bound to framework
S2	−295, −228^a^ , −162	0.06	3.65	Co-NO-(NH_3_)_3+x_ + (4-x)NH_3,gas_ (x = 1 or 2) Nearly unbound from framework
T	-95, -28^a^ , +38	0.02	4.43	Co-NO-(NH_3_)_3_ + 4NH_3,gas_ Not bound to framework

^a^Most intense peak

Figures 6 and 7 show power spectra obtained for S1 and S2 trajectories simulating the behavior of Co(II)-NO adducts in ammonia-excess conditions (dipole moment autocorrelation function was not calculated, thus line shapes are not available). Table 6 summarizes average characteristics inferred from Figs 6 and 7 together with anharmonic stretching frequencies extracted from the three MD simulations carried out for the model of fully ammonia saturated system: S1, S2 and T states of {Co^2+^–NO–(NH_3_)_3_ + 4NH_3_} adduct, encapsulated in a CHA chamber. In both types of singlet simulations the frequency of 1602 cm^−1^, shifted by −228 cm^−1^ with respect to NO frequency calculated for free NO in a periodic box (equal to 1830 cm^−1^), dominates the spectrum, surprisingly well matching the red-shift of -226 cm^−1^ obtained by Hessian diagonalization for a small, T1-based cluster model. Indeed, the analysis of S1-type trajectory revealed that 3 NH_3_ ligands remained stably bonded to the cobalt center (still weakly linked to the framework), even in excess ammonia condition (see the inset in Fig. 6). In the S2-type simulation the same frequency dominated the NO spectrum, although particular forms of adducts contained predominantly the complexes with four (occasionally five) NH_3_ ligands, much weaker bound to the framework (cf. Fig. 7). We are analyzing the S2-type spectrum in more detail in the next paragraph, jointly with other [Co(II)–NO](NH_3_)_n_ forms. Nevertheless, this result may be taken as strong support for our former suggestion that the most red-shifted peak measured in ammonia-pretreated Co(II)-MOR zeolite originates from [Co(II)–NO]–(NH_3_)_3_ adducts [23].

## Summary and conclusions

Table 7 sets together the values of NO stretching frequencies, measured by FTIR spectroscopy for Co(II)MOR zeolite [22] and calculated within several schemes invoked by us in this work and in our former studies. Dominating peaks derived from MD simulations yield several discrete frequencies that correspond best to actual IR spectra: this indicates that MD simulations should reproduce experimental conditions reasonably well. Therefore, we have scaled up all calculated anharmonic frequencies by uniform shifting by 45 cm^−1^, i.e., by the difference between the frequency calculated by MD for NO molecule in a periodic box (1830 cm^−1^) and that measured for the gas phase NO (1875 cm^−1^). Scaled values (v**_per_ in column 3 of Table 7) directly compare with the frequencies measured for MOR zeolite (column 2). Columns 5 and 6 compare also the relevant red-shifts of NO frequencies extracted from MD simulations and from various cluster models, described in the section on cluster calculations. These serve both to illustrate the relevance of the used cluster models and to support the interpretation of various adduct forms inferred from MD trajectories.

**Table 7:** Comparison of anharmonic frequencies calculated for experimentally relevant models (shifted up by 45 cm^−1^, ν**_per_) with frequencies measured by FTIR for MOR zeolite (*ν* ^MOR^_NO_); frequency shifts (∆*ν*_NO_) calculated for clusters with nNH_3_ included for comparison

Experiment		Calculations			
*ν* ^MOR^_NO_		ν**_per_		∆*ν*_NO_^per^	∆*ν*_NO_^clust^
NH_3_ deficiency	1765,	2-NH_3_ T	1847	-28	+6 (2)_T1_
	1745	2-NH_3_ S	1713	-162	-186 (2)_T12_
NH_3_ surplus		7-NH_3_ S1	1647	-228	-226 (3) _T1_
					-217 (3)_T18_
	1600^a^	7-NH_3_ S2	1580^a^	-295^a^	-226 (3) _T1_
					-217 (3)_T18_
	1650		1647	-228	-188 (4)_T18_
	1730		1713	-162	-166 (5)_gas_
	-	7-NH_3_ T	1780	-95	-80 (3) _T1_
			1847	-28	-56 (2) _T1_
			1913	+38	-

^a^Weak experimental features assigned to other forms of strong NH_3_ adsorption in excess of ammonia conditions [34, 35]

General comparison of frequencies derived from MD simulations for periodic models (shifted uniformly up by the difference between the NO stretching frequency measured in gas phase and that calculated by MD) with the IR NO frequency measured for a real zeolite (being general target of our modeling) points to a very good agreement between the experiment and theory. We take this good reproduction of experimental NO spectrum as strong support for the MD protocol adopted in this work, even if it does not fully comply with the strict requirements expected from quantitative simulations. We are also aware that our simulations concerned the chabasite, while IR spectra were registered for other zeolite types. Therefore, we do not attempt to quantitatively reproduce properties of the system with exactly defined structure and composition but would rather search for systematic trends in hope to uncover physical reasons for measured and calculated phenomena. Nevertheless, by applying periodic models and MD simulations in realistic conditions, we have made one more step towards actual zeolites.

Along these lines, it seems that our new results enrich and modify the interpretation of IR spectra (calculated for small cluster models), and strongly support the suggestions that the forms of {Co(II)–NO} adducts comprising three or four ammonia ligands dominate IR spectra taken in ammonia-saturation conditions while the forms with two ammonia ligands prevail in the case when saturation of a Co^2+^-exchanged zeolite by ammonia is still incomplete. The analysis of MD trajectories in terms of cobalt interactions with ammonia donors (measured by average number and distance of NH_3_ ligands) and with electron-rich bridging oxygens (measured by the coordination number and the distance from the framework) indicate that both are of comparable importance. The comparison of two singlet trajectories representing ammonia-rich conditions shows that two situations are seemingly equivalent with respect to the NO activation (as indicated by nearly identical red-shift of NO frequency): Co(II)–NO core either saturated by three ammonia donors and still well supported by framework oxygens, or that with four ammonia donors and still acting but much weaker influence of the framework (cf. S1 and S2 trajectories). However, the case with the coordination of Co fully saturated by five ammonia and NO ligands, leaving no space for framework support, substantially decreases the red-shift of NO frequency (by roughly 70 reciprocal centimeters), which points to a very important role of the framework as a generalized donor. This situation complies with the behavior of Cu(I) sites in SSZ-13 zeolite under NH_3_-SCR reaction conditions: the difference seems to come from various coordination preferences between Co^II^ and Cu^I/II^ cations.

In conclusion, the critical dependence of the activation ability of Co(II) centers in zeolites towards NO upon their donor properties is again confirmed by relating the IR-measured and calculated fingerprint of the NO activation (red-shift of NO stretch) to the analysis of electron transfer processes (reinforced both by electron-donating co-ligands like NH_3_ and zeolite framework). However, it should be stressed here that strongly bound ligands may effectively compete with the interaction of the center with the electron-rich framework, and a balance must be observed to keep optimal electron inflow and activation ability of the site. Transition metal centers have long been known to act as the junction between the electron reservoir (generalized catalyst support and additional donors) and the substrate (here: NO), performing roles of “electron sink” and “electron tap” [36]. In this respect, speciation of the electronic state of the center may be important, which may explain strong dependence of electron donation processes on the spin of the system: this work implicitly confirms that effective electron transfer channels are apt to open for the singlet spin state of the Co(II)–NO adduct and to close for the triplet state. Here, ammonia ligands may act not only as good electron donors but also as spin-dumping ligands.

## Electronic supplementary material

Below is the link to the electronic supplementary material.

ESM 1 Electronic Supplementary Information (ESI) is available on-line containing power spectra for velocity autocorrelation function and Tables with geometrical parameters and shifts of anharmonic frequencies for relevant studied adducts of Co(II), NO and NH_3_. (PDF 411 kb)
