# Supplementary material for: The dependence on ammonia pretreatment of N−O activation by Co(II) sites in zeolites: a DFT and ab initio molecular dynamics study
Source: J Mol Model. 2017 Apr 13;23(5):160. doi: 10.1007/s00894-017-3322-z (PMC5393292; doi:10.1007/s00894-017-3322-z)
Supplement: Supplementary file 1 — Electronic Supplementary Information (ESI) is available on-line containing power spectra for velocity autocorrelation function and Tables with geometrical parameters and shifts of anharmonic frequencies for relevant studied adducts of Co(II), NO and NH3. (PDF 411 kb) [file 894_2017_3322_MOESM1_ESM.pdf]

The dependence of N−O activation by Co(II) sites in zeolites upon ammonia pretreatment: DFT and ab initio Molecular Dynamics study.

E. Broclawik, K. Góra-Marek, M. Radoń, T. Bučko and A. Stępniewski

Electronic Supplementary Information

Table S1. Selected geometrical parameters of lowest-energy optimized structures (from 50 random snapshots selected from MD trajectories), cobalt distance from the 3T framework plane PD, its coordination number LK and the shift of anharmonic NO stretching frequency Δv^P for up to two-ammonia adducts over the six-ring (in singlet (S) and triplet (T) states); averages over MD runs are given for each trajectory in parentheses.

	$0NH_3$(S)	$0NH_3$(T)	$1NH_3$(S)	$1NH_3$(T)	$2NH_3$(S)	$2NH_3$(T)
			Angle (°)			
Co-N-O	129.1	159.5	164.6	151.2	125.4	167.9
			Bond distances (Å)			
Co-O	1.97; 2.00; 2.04; 2.07<br>(1.98; 2.00; 2.10; 2.11)	2.03; 2.06; 2.14; 2.16<br>(2.04; 2.07; 2.19; 2.22)	1.91; 1.92; 3.70; 3.80<br>(1.90; 1.96; 3.88; 4.33)	2.09; 2.10; 2.17; 3.27<br>(1.99; 2.17; 2.75; 3.34)	1.98; 2.02; 3.52; 3.77<br>(1.99; 2.07; 3.70; 4.09)	2.00; 2.51; 4.02; 4.22<br>(2.00; 2.67; 3.93; 4.62)
Co-NO	1.75 (1.76)	1.69 (1.71)	1.62 (1.62)	1.71 (1.70)	1.76 (1.77)	1.68 (1.69)
N-O	1.17 (1.18)	1.16 (1.16)	1.16 (1.16)	1.17 (1.17)	1.19 (1.19)	1.16 (1.16)
Co-NH_3	—	—	1.93 (1.95)	2.08 (2.11)	1.94; 1.95<br>(1.96; 1.97)	2.03; 2.08<br>(2.02; 2.09)
		Co-Al and distances from 3T-plane (Å), coordination number				
Co-Al	2.89; 2.91<br>(2.94; 2.94)	2.96; 2.98<br>(3.01; 3.02)	2.73; 4.60<br>(2.71; 4.88)	2.98; 3.42<br>(3.19; 3.49)	2.79; 4.46<br>(2.81; 4.71)	2.94; 4.95<br>(2.98; 5.07)
PD_{Co}	0.74 (0.75)	0.98 (0.95)	1.74 (1.82)	1.30 (1.45)	1.86 (1.92)	2.26 (2.29)
LK	2.27 (2.01)	2.02 (1.78)	1.43 (1.39)	1.58 (1.45)	1.27 (1.22)	0.91 (0.88)
		Anharmonic shift of NO frequency (cm^{-1})				
Δv^P	-95	+39	+106	+39, -28	-161	+38, -29

Table S2. Selected geometrical parameters of lowest-energy optimized structures (from 50 random snapshots selected from MD trajectories), cobalt distance from the 3T framework plane PD, its coordination number LK and the shift of anharmonic NO stretching frequency Δv^{P} for one- to three-ammonia adducts over the eight-member ring (in singlet (S) and triplet (T) states); averages over MD runs are given for each trajectory in parentheses.

	$1NH_3$(S)	$1NH_3$(T)	$2NH_3$(S)	$2NH_3$(T)	$3NH_3$(S)	$3NH_3$(T)
			Angle (°)			
Co-N-O	161.6	176.4	178.2	141.9	121.7	149.5
			Bond distances (Å) (Å)			
Co-O	1.92; 1.94 (1.93; 1.96)	1.92; 2.00 (1.95;2.06)	2.09; 2.10 (2.11;2.12)	2.09; 2.14 (2.11;2.13)	2.08; 2.46 (2.13;2.45)	2.16; 2.37 (2.22;2.41)
Co-NO	1.62 (1.64)	1.68 (1.69)	1.61 (1.62)	1.71 (1.72)	1.78 (1.78)	1.69 (1.71)
N-O	1.16 (1.17)	1.15 (1.16)	1.17 (1.17)	1.17 (1.18)	1.20 (1.20)	1.17 (1.17)
Co-NH_3	1.93 (1.95)	2.05 (2.07)	1.96; 1.98 (1.97;2.00)	2.03; 2.06 (2.04;2.10)	1.97; 1.98; 2.00 (1.98; 1.99; 2.02)	2.09; 2.16; 2.17 (2.12; 2.18; 2.18)
			Co-Al and distances from 3T-plane (Å), coordination number			
Co-Al	2.80; 5.39 (2.81; 5.51)	2.77; 5.77 (2.81;5.76)	2.90; 5.61 (2.93;5.62)	2.87; 5.69 (2.91;5.58)	3.02; 5.43 (3.03;5.59)	3.01; 5.57 (3.06;5.65)
PD_{Co}	0.62 (0.64)	0.80 (0.65)	0.94 (0.91)	1.02 (0.82)	1.09 (1.16)	1.17 (1.23)
LK	1.29 (1.28)	1.22 (1.23)	1.01 (0.99)	0.97 (1.01)	0.80 (0.78)	0.77 (0.73)
			Anharmonic shift of NO frequency (cm^{-1})			
Δv^{NO}_{P}	+38	+106	+39	-28, -95	-228	-28

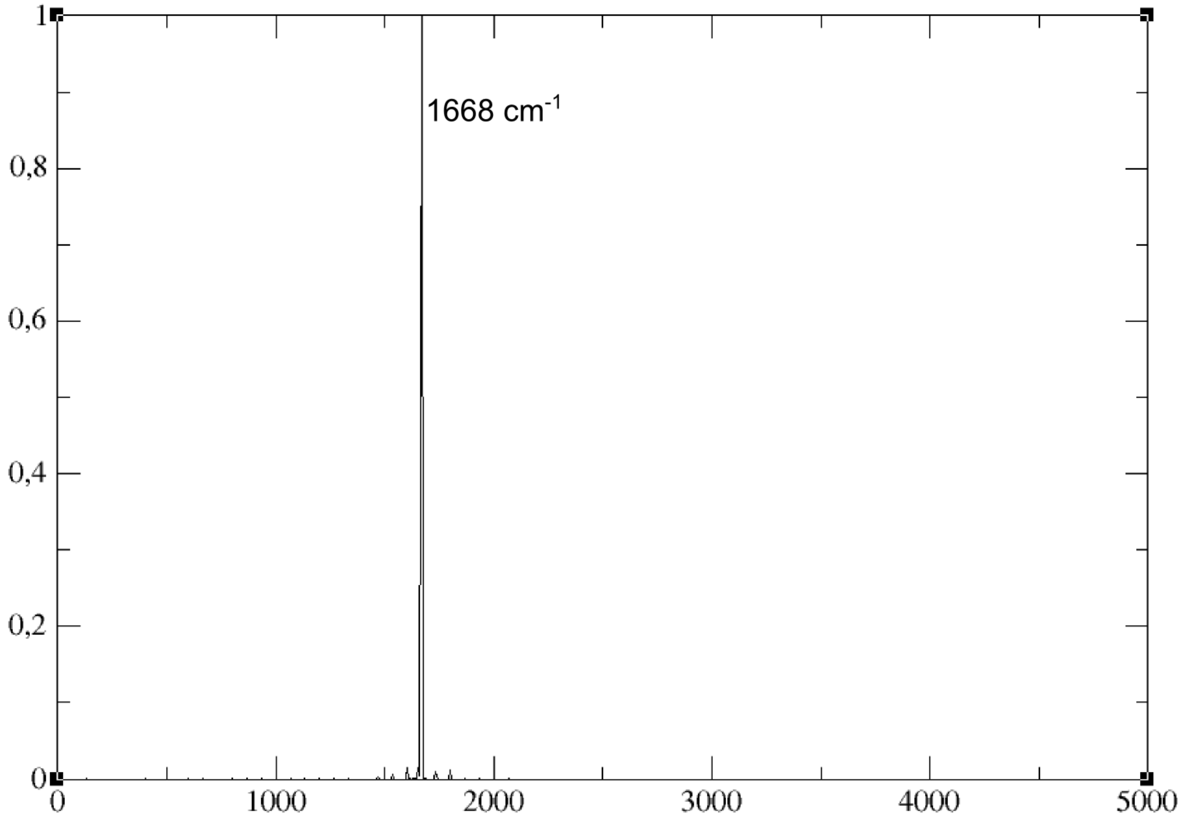

Fig, S1. Power spectrum from velocity autocorrelation function filtered with respect to N_{NO} and O_{NO} coordinates for singlet simulation for the {Co^{2+}-NO-$(NH_3)_2$} system anchored in 6-member position; frequencies in cm^{-1}, intensity in arbitrary units.

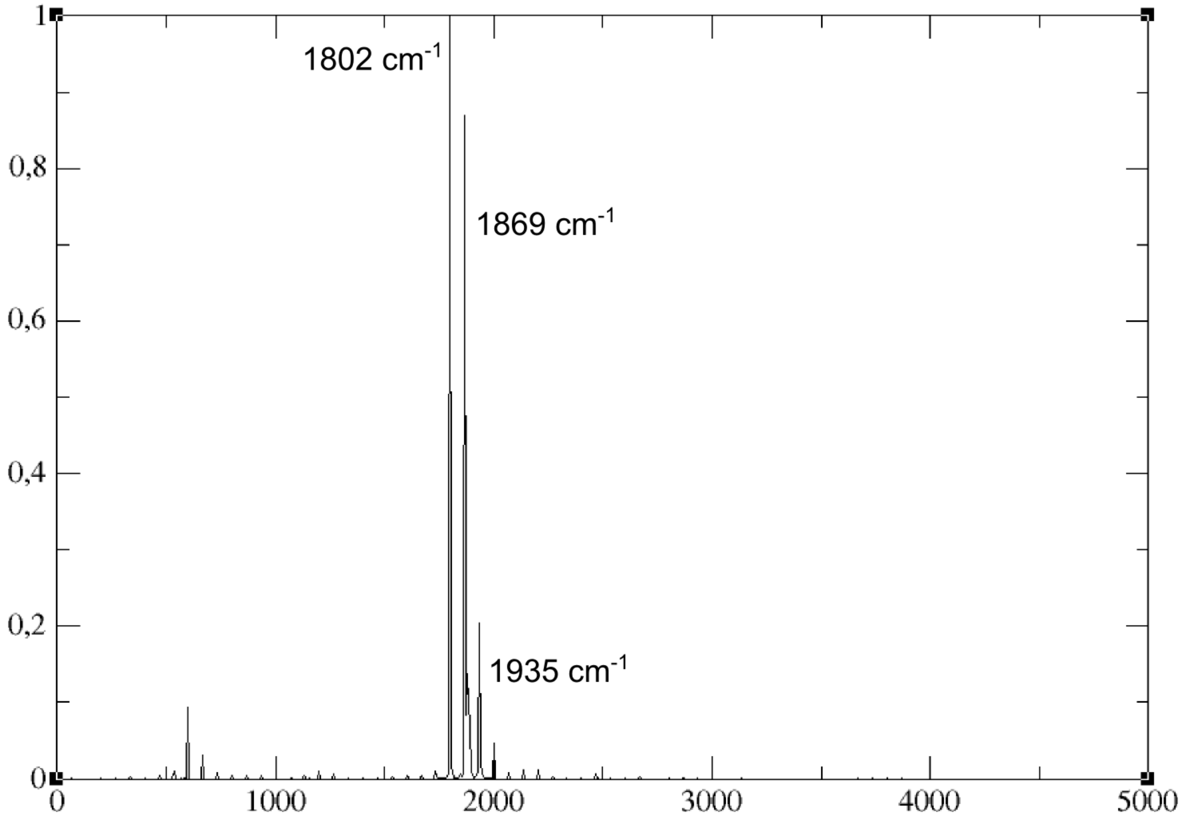

Fig, S2. Power spectrum from velocity autocorrelation function filtered with respect to N_{NO} and O_{NO} coordinates for triplet simulation for the $\{Co^{2+}\text{-NO-}(NH_3)_2\}$ system anchored in 6-member position; frequencies in cm^{-1}, intensity in arbitrary units.

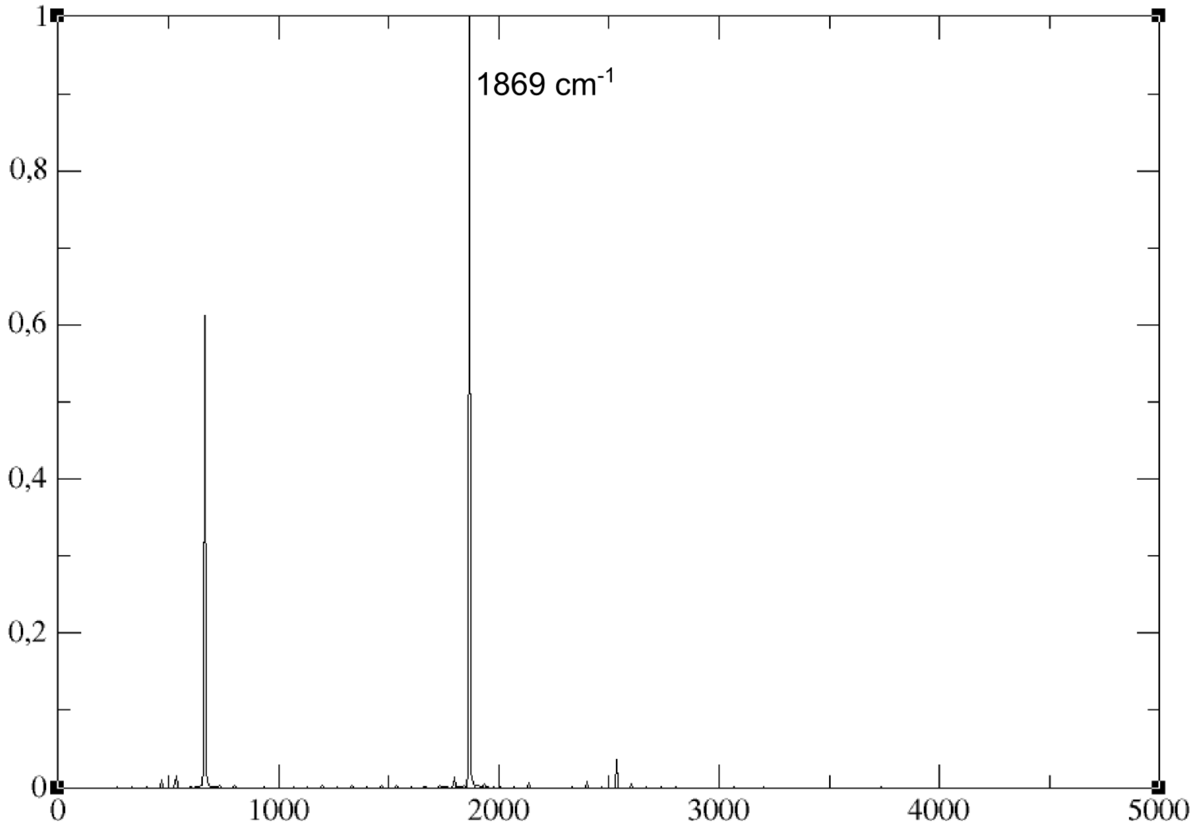

Fig, S3. Power spectrum from velocity autocorrelation function filtered with respect to N_{NO} and O_{NO} coordinates for singlet simulation for the $\{Co^{2+}$-NO-$(NH_3)_2\}$ system anchored in 8-member position; frequencies in cm^{-1}, intensity in arbitrary units.

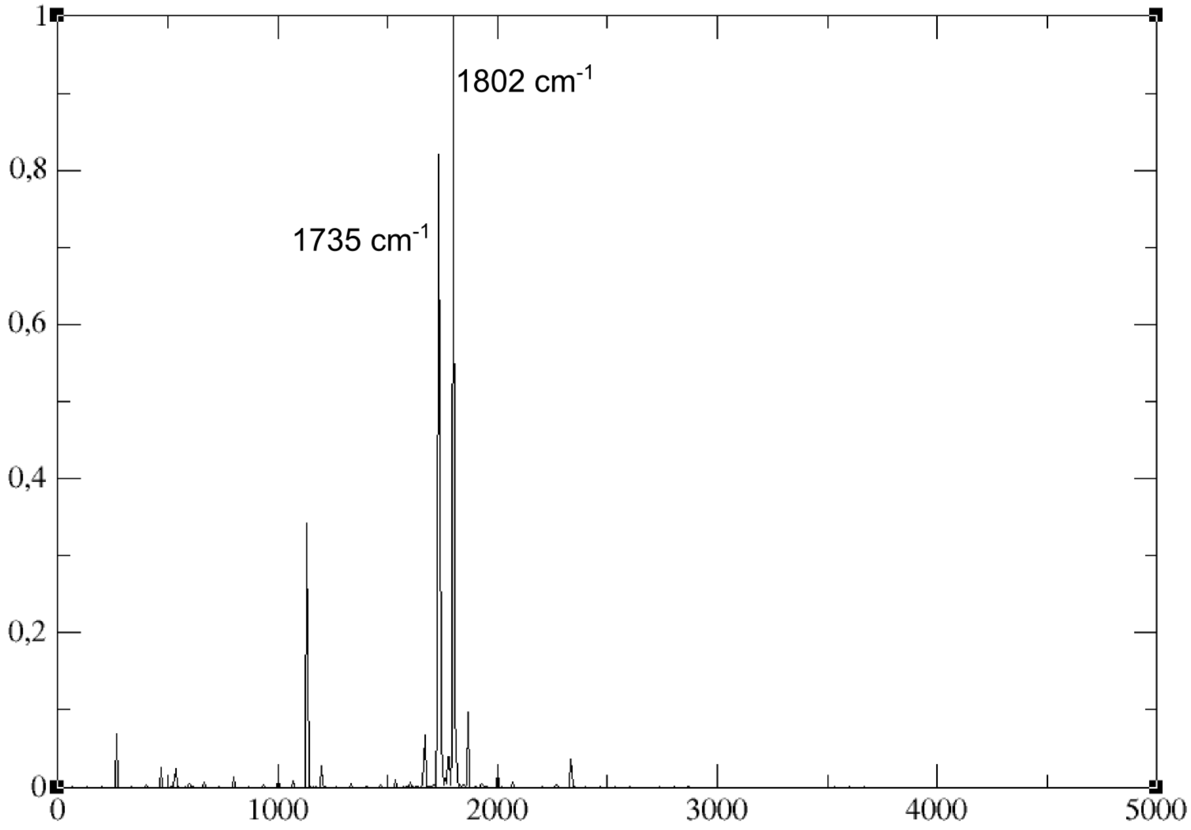

Fig, S4. Power spectrum from velocity autocorrelation function filtered with respect to N_{NO} and O_{NO} coordinates for triplet simulation for the $\{Co^{2+}$-NO-$(NH_3)_2\}$ system anchored in 8-member position; frequencies in cm^{-1}, intensity in arbitrary units.

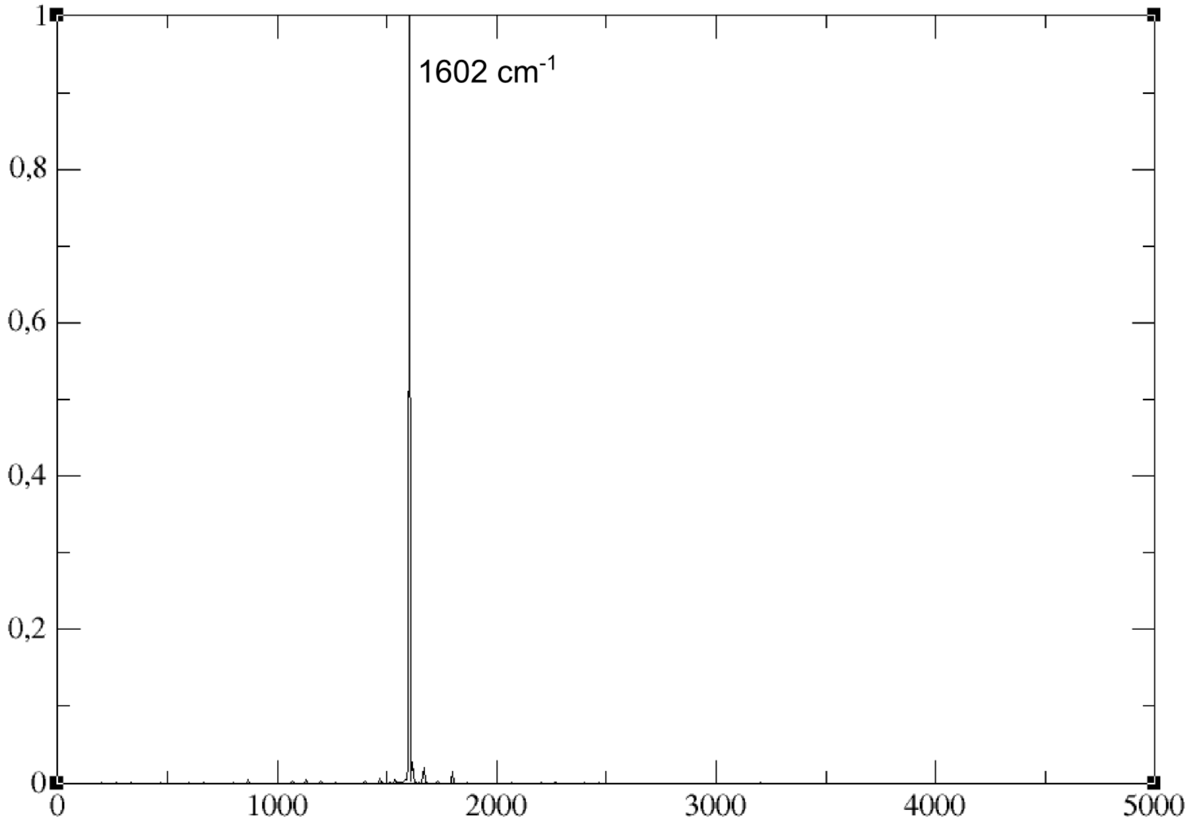

Fig, S5. Power spectrum from velocity autocorrelation function filtered with respect to N_{NO} and O_{NO} coordinates for singlet simulation for the {Co^{2+}-NO-$(NH_3)_3$} system anchored in 8-member position; frequencies in cm^{-1}, intensity in arbitrary units.

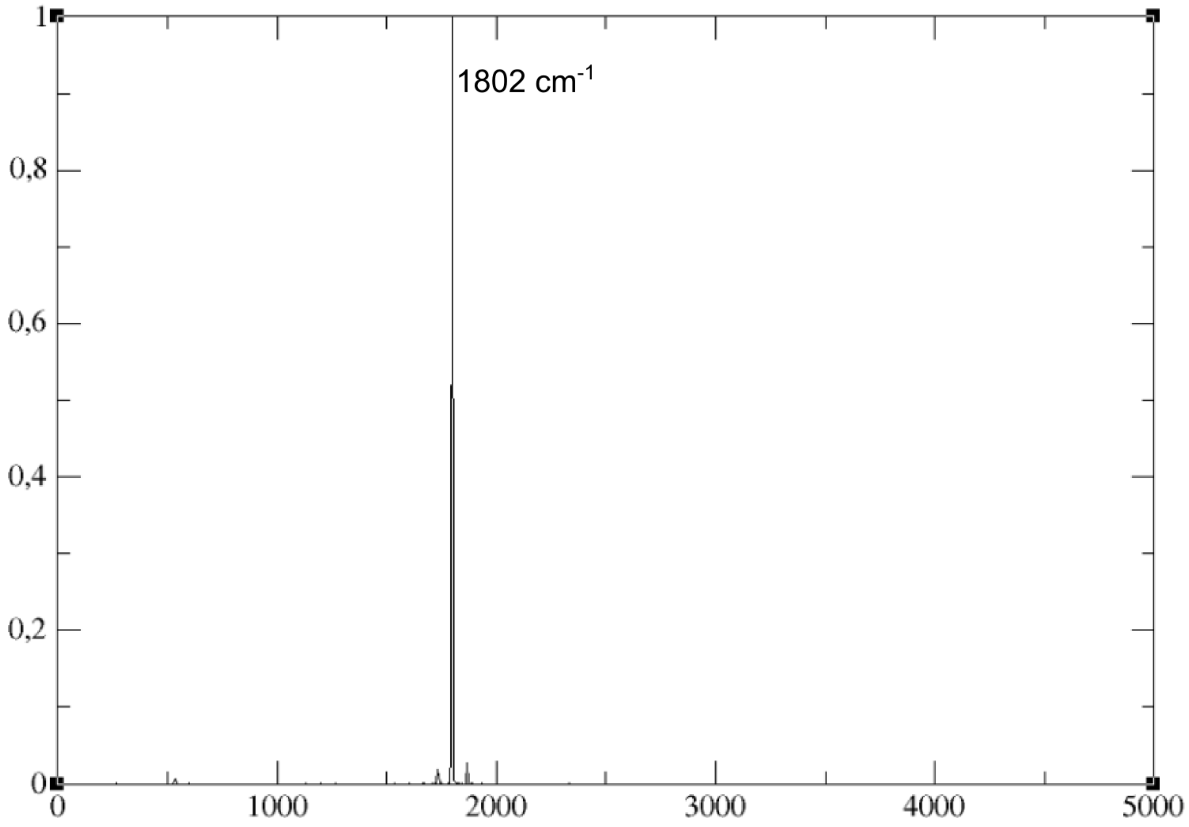

Fig, S6. Power spectrum from velocity autocorrelation function filtered with respect to N_{NO} and O_{NO} coordinates for triplet simulation for the {Co^{2+}-NO-$(NH_3)_3$} system anchored in 8-member position; frequencies in cm^{-1}, intensity in arbitrary units.

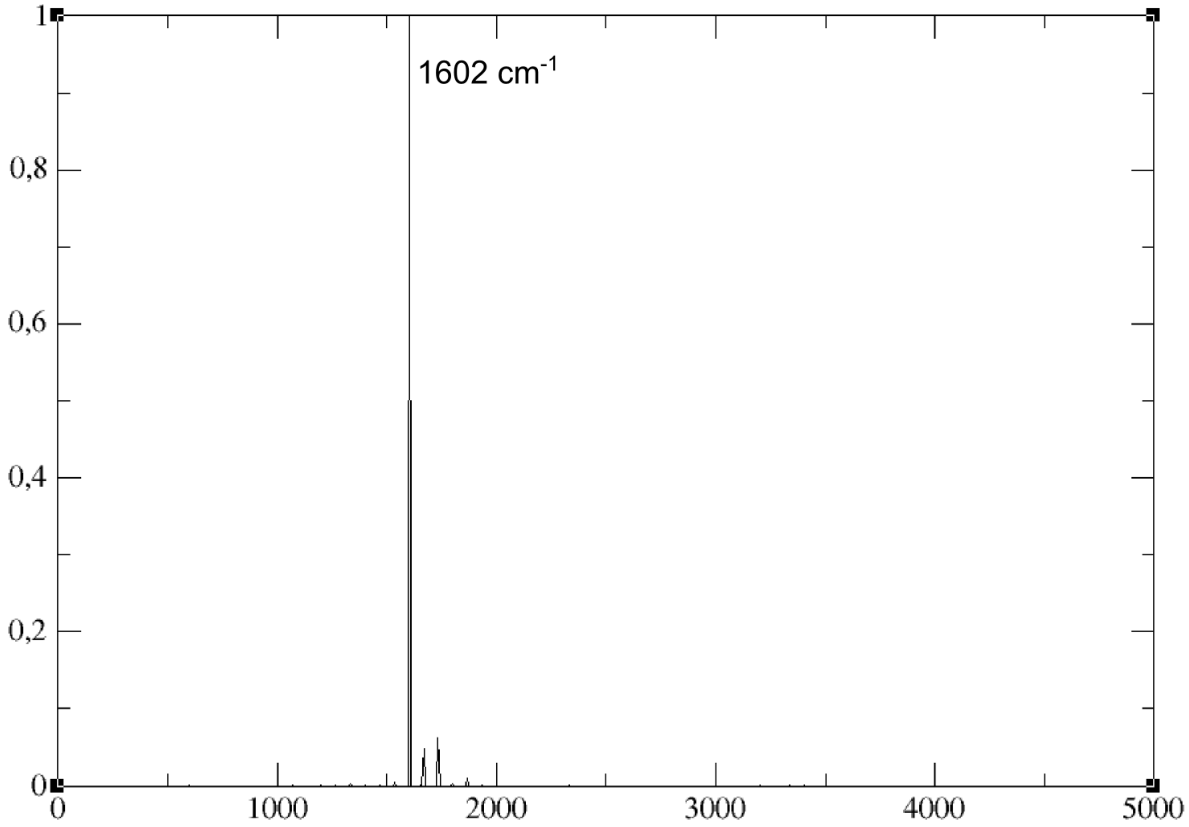

Fig, S7. Power spectrum from velocity autocorrelation function filtered with respect to N_{NO} and O_{NO} coordinates for S1-type singlet simulation for the $\{Co^{2+}$-NO-$(NH_3)_3 + 4NH_3\}$ system; frequencies in cm^{-1}, intensity in arbitrary units.

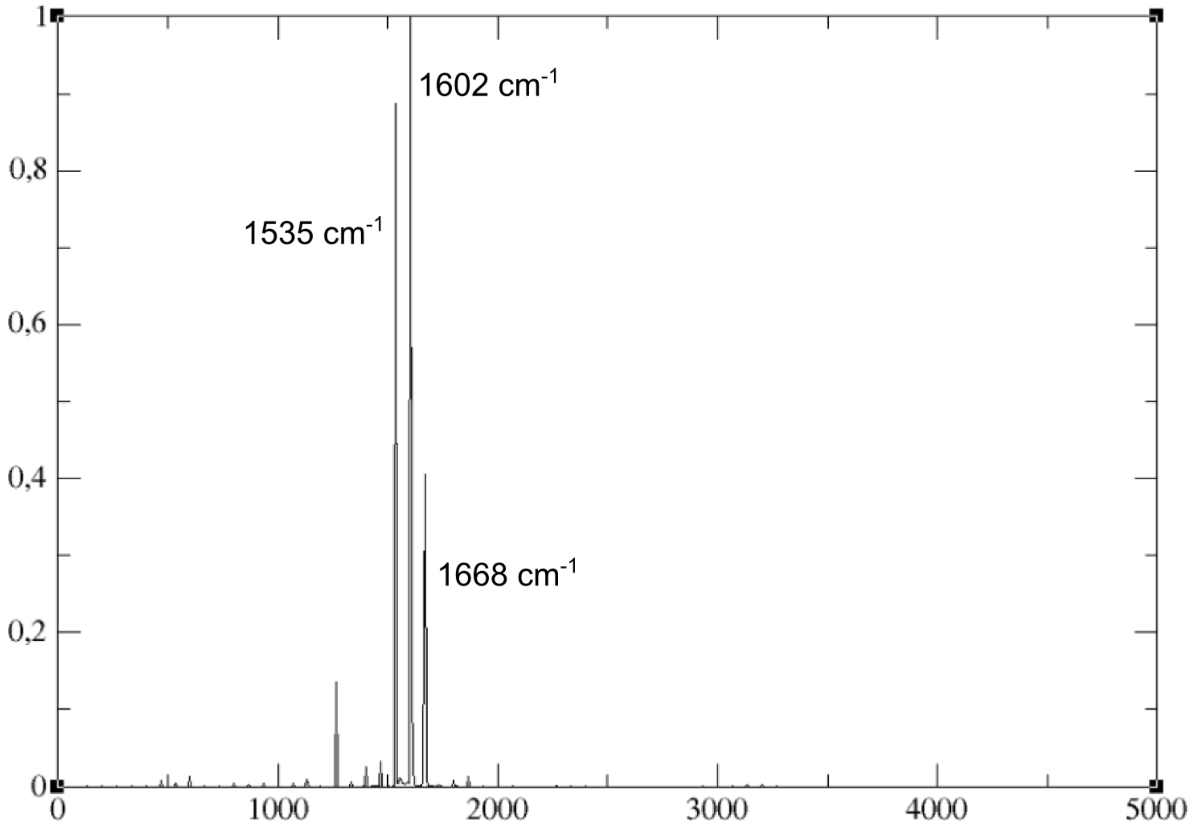

Fig, S8. Power spectrum from velocity autocorrelation function filtered with respect to N_{NO} and O_{NO} coordinates for S2-type singlet simulation for the $\{Co^{2+}\text{-NO-}(NH_3)_3 + 4NH_3\}$ system; frequencies in cm^{-1}, intensity in arbitrary units.
